# Outlook on Chronic Venous Disease Treatment: Phytochemical Screening, In Vitro Antioxidant Activity and In Silico Studies for Three Vegetal Extracts

**DOI:** 10.3390/molecules28093668

**Published:** 2023-04-23

**Authors:** Andreea Roxana Ungureanu, Carmen Lidia Chițescu, Emanuela Alice Luță, Alina Moroșan, Dan Eduard Mihaiescu, Dragoș Paul Mihai, Liliana Costea, Emma Adriana Ozon, Ancuța Cătălina Fița, Teodora Dalila Balaci, Rica Boscencu, Cerasela Elena Gîrd

**Affiliations:** 1 Faculty of Pharmacy, Carol Davila University of Medicine and Pharmacy, Traian Vuia 6, 020956 Bucharest, Romania; liliana.costea@drd.umfcd.ro (L.C.); emma.budura@umfcd.ro (E.A.O.); catalina.fita@umfcd.ro (A.C.F.); teodora.balaci@umfcd.ro (T.D.B.); rica.boscencu@umfcd.ro (R.B.); cerasela.gird@umfcd.ro (C.E.G.); 2 Faculty of Medicine and Pharmacy, Dunărea de Jos University of Galați, A.I. Cuza 35, 800010 Galați, Romania; carmen.chitescu@ugal.ro; 3 Faculty of Chemical Engineering and Biotechnologies, University of Politehnica, Gheorghe Polizu 1-7, 011061 Bucharest, Romania; alina.morosan@upb.ro (A.M.); dan.mihaiescu@upb.ro (D.E.M.)

**Keywords:** chronic venous disease, antioxidant activity, *Sophorae flos* extract, *Ginkgo bilobae folium* extract, *Calendulae flos* extract, phytochemical composition, molecular docking, matrix metalloproteinase

## Abstract

Chronic venous disease is one of the most common vascular diseases; the signs and symptoms are varied and are often neglected in the early stages. Vascular damage is based on proinflammatory, prothrombotic, prooxidant activity and increased expression of several matrix metalloproteinases (MMPs). The aim of this research is preparation and preliminary characterization of three vegetal extracts (*Sophorae flos*-SE, *Ginkgo bilobae folium*-GE and *Calendulae flos*-CE). The obtained dry extracts were subjected to phytochemical screening (FT-ICR-MS, UHPLC-HRMS/MS) and quantitative analysis (UHPLC-HRMS/MS, spectrophotometric methods). Antioxidant activity was evaluated using three methods: FRAP, DPPH and ABTS. More than 30 compounds were found in each extract. The amount of flavones follows the succession: SE > GE > CE; the amount of phenolcarboxylic acids follows: SE > CE > GE; and the amount of polyphenols follows: SE > GE > CE. Results for FRAP method varied as follows: SE > CE > GE; results for the DPPH method followed: SE > GE > CE; and results for ABTS followed: SE > GE > CE. Strong and very strong correlations (appreciated by Pearson coefficient) have been observed between antioxidant activity and the chemical content of extracts. Molecular docking studies revealed the potential of several identified phytochemicals to inhibit the activity of four MMP isoforms. In conclusion, these three extracts have potential in the treatment of chronic venous disease, based on their phytochemical composition.

## 1. Introduction

Current guidelines define chronic venous disease as any long-term morphological and functional abnormality of the venous system. Pathogenesis results from hemodynamic abnormality (venous reflux and venous hypertension) caused by valve incompetence and/or venous flow obstruction and/or muscle pump incapacity. The pathological cascade is based on a multimodal mechanism. There is an imbalance in the activity of endothelial cells on hypoxic background (NO and PGI2 levels decrease), and leukocyte infiltration begins under the signaling given by ICAM-1, VCAM-1 and selectins (P-, L-, E-). The damage involves a high level of pro-inflammatory mediators which stimulate the NF-kB pathway (TNF-α, IL-6, IL-8) and the induction of metalloproteinases, in parallel with increased level of prothrombotic agents causing platelets aggregation (vWF, PAI1) as well as the acceleration of oxidative processes (stimulation of NADPH oxidase and generation of free radicals). In summary, these metabolic perturbances at the cellular level are attributed to increased proinflammatory, procoagulant and prooxidant activities. From a clinical point of view, chronic venous disease causes varied signs and symptoms depending on the evolutionary stage: feeling of heavy legs, paresthesia, edema, telangiectasias, varicose veins, hyperpigmentation and chronic ulcerations [1,2,3].

In chronic venous disease, the expression and activity levels of matrix metalloproteinases (MMPs, Zn^2+^-dependent endopeptidases involved in extracellular matrix degradation) are disrupted. Some studies have highlighted high levels of MMPs (MMP-1, MMP-2, MMP-3, MMP-9) with different locations of venous layers. MMP-1 was detected in high levels in fibroblasts, while MMP-9 was measured in endothelial cells and adventitia. Other studies showed increased levels of MMP-2 in all venous layers and of MMP-1 and MMP-3 in the intima and media of varicose veins. Higher expressions were also observed for pro-MMP-9 (precursor) and MMP-9. MMP-9 was found throughout the venous wall in both varicose and normal veins, but an overexpression was observed in the smooth muscle layer of varicose veins. There have also been studies that either noticed decreases in the levels of MMPs in varicose veins or no changes at all. The variability may be due to the examination of different areas of the venous path. Certain areas are atrophic; matrix degradation occurs; and certain areas are hypertrophic with matrix accumulation hindering the contractility of the vascular smooth muscles and conducting to venous dilatations (in addition, MMP-2 induces vasorelaxation through hyperpolarization and activation of BK_Ca_ channels) [4,5,6,7,8].

Medicines used for chronic venous disease have been grouped into a general category called phlebotonics [9]. Later, the classification was divided in two categories: venoactives (they decrease capillary permeability and reduce the release of pro-inflammatory mediators) and non-venoactives (they reduce leukocyte activity and platelet aggregation). The main physiopathological objectives of treatment are: 1. increasing the venous tone and 2. reducing capillary permeability and fragility. The list of objectives can be extended as follows: reduction of microcirculatory problems, correction of hemorheological deviations, improvement of parietal fibrinolytic activity and reduction of venous ulceration progression [2,3].

Natural sources comprise a variety of phytochemical classes that may be used in the treatment of chronic venous disease. The main pharmacological treatment consists of purified and micronized flavonoid fractions (MPFF), being included in the therapeutic guideline under the IIa recommendation class, with the highest level of evidence (level A). They demonstrated cumulative effectiveness on several pathological aspects: venotonic action (inhibits noradrenaline metabolism), antioxidant and anti-inflammatory action, a protective effect against valvular damage induced by inflammatory factors (delays venous reflux), inhibition of leukocyte activation, migration and adhesion and increased capillary resistance [2,3,9,10,11,12,13].

The vegetal product *Sophorae flos* is known for its content of flavonoids: rutin, isoquercetin, kaempferol, isorhamnetol, genistein and pratensin and of terpenes: sophoradiol, betulinol, soyasaponin and phaseoside. It is a source of flavonoids, especially of rutin. The chemical composition defines its anti-inflammatory, antioxidant, antitumor and antibacterial effects. Its vasoprotective action, useful in chronic venous disease, can be explained due to effects exerted by flavonoid content: anti-inflammatory (inhibition of the p38 MAPK signaling pathway, inhibition of leukocyte infiltrates, decrease in NF-kB activation, inhibition of matrix-metalloproteinases), antioxidant (quenching hydroxyl and superoxide free radicals, chelation of metal ions, increase of SOD activity) and antiplatelet effect (inhibition of aggregation induced by TXA2 pathway, decreasing Ca^2+^ free at platelet level) [14,15,16,17].

Another source of flavonoids is *Ginkgo bilobae folium* which contains derivatives of quercetol, kaempferol, isorhamnetol, myricetol, bilobalide, ginkgolides and ginkgolic acids. The main effect is neuroprotective, being indicated in neurodegenerative diseases, but it is also useful in arteriopathy of the lower limbs having an arterial and venous vasomodulatory role (improves blood flow, favors tissue perfusion, increases resistance to hemolysis). The anti-inflammatory action is characterized both by lowering the level of inflammatory markers (inhibits COX-2, TNF-α) and by reducing transcription at the NF-kB pathway. It hinders the effects of PAF (platelet aggregation and clot formation) while also preventing cytokine-stimulated leukocyte adhesiveness. The antioxidant activity complements actions previously mentioned for describing the multimodal effect and is explained by the neutralization of reactive species protecting cells from oxidative damage [14,18,19,20].

The most advanced phases of venous insufficiency are characterized by chronic ulcerations. The onset occurs through microcirculatory destruction followed by fluid extrusion conducting to edema. Decreases oxygen supply to adjacent tissues causes necrosis and deep ulceration. Natural product *Calendulae flos* is a source of phytochemical compounds with wound-healing, anti-inflammatory, antioxidant and anti-edematous effects: flavonoids (derived from quercetol and isorhamnetol), triterpenes (calendulosides) and coumarins (esculetol, umbelliferone). Ethanol extract of *Calendula* modulates inflammatory activity (inhibits COX-2, LOX-5 and C3-covertase), activates fibroblast migration (by PI3K pathway) and inhibits collagenase activity. At keratinocyte level, it favors their migration and proliferation, supporting the re-epithelialization process. Additionally, ethanolic extract (70%) in gel formulation increased the level of collagen and hydroxyproline (as indicator of collagen synthesis) at lesion sites [21,22,23,24,25,26].

In this context, the aim of the current research was to obtain and preliminarily characterize the plant extracts with therapeutic potential in the management of chronic venous disease, in the perspective of their integration in complex pharmaceutical forms. Moreover, a molecular docking study was performed to investigate the potential of the identified phytoconstituents to inhibit the activity of four MMPs, due to their involvement in the pathophysiology of chronic venous disease.

## 2. Results

### 2.1. Preparation of Vegetal Extracts

**Figure 1:**
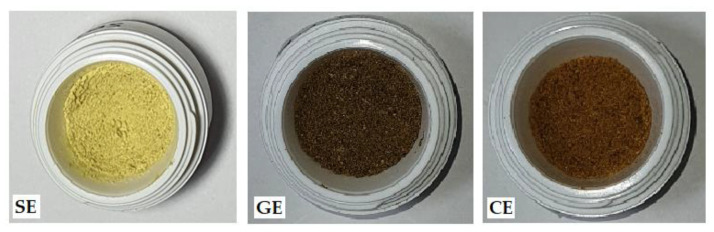
Dry extracts obtained after lyophilization (SE—*Sophorae flos* extract, GE—*Ginkgo bilobae folium* extract, CE—*Calendulae flos* extract).

The obtained extracts were dry powders with organoleptic properties similar to their source (Figure 1). *Sophorae flos* extract (SE) is green-yellow; *Ginkgo bilobae folium* extract (GE) is green-brown; and *Calendulae flos* extract (CE) is orange-brown.

### 2.2. Qualitative Analysis FT-ICR-MS (Fourier-Transform Ion-Cyclotron-Resonance High-Resolution Mass Spectrometry)

Following FT-ICR-MS analysis, ESI+ and ESI–, we identified eight compounds for *Sophorae flos* extract (Table 1), 12 compounds for *Ginkgo bilobae folium* extract (Table 2) and 10 compounds for *Calendulae flos* extract (Table 3). Spectrograms are presented in Supplementary Materials Figures S1–S28.

**Figure 2:**
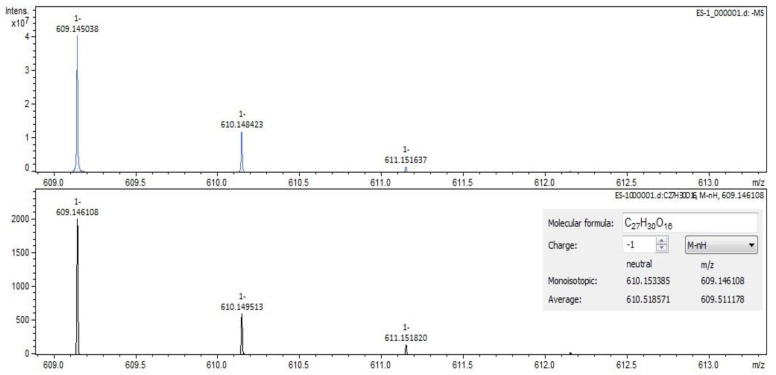
Rutin identification in SE–spectrogram (ESI–,water sample).

**Figure 3:**
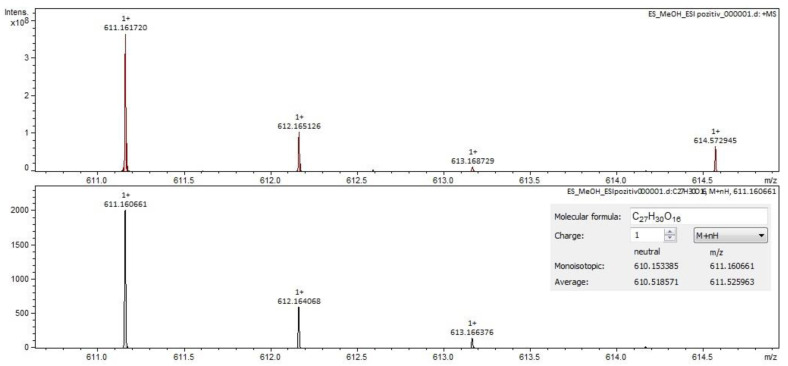
Rutin identification in SE–spectrogram (ESI+, methanol sample).

For *Sophorae flos* extract, rutin (Figure 2 and Figure 3) and quercetin were identified by both ESI+ and ESI– regardless of the solvent of the sample. Isorhamnetin, isoquercitrin and kaempferol were identified by ESI+ in both solvents and by ESI– only in methanol samples. Sophoricoside was identified only by ESI+ in both solvents. Kaikasaponin I was identified by both ESI+ and ESI– in the methanol sample, and kaikasaponin III was identified only by ESI+ in the methanol sample.

In *Ginkgo bilobae folium* extract, rutin and bilobalide were identified by both ESI+ and ESI- for both solvents. Quercetin was identified by ESI+ in water sample and methanol sample but by ESI– only in the water sample. Ginkgolides A, B and C were identified by ESI+ in both solvents, and by ESI– only ginkgolide B was identified in the methanol sample. Kaempferol, isoquercitrin, isorhamnetin and catechin were identified by ESI+ in both solvents. Bilobetin and caffeic acid were found by ESI+ in methanol sample.

**Figure 4:**
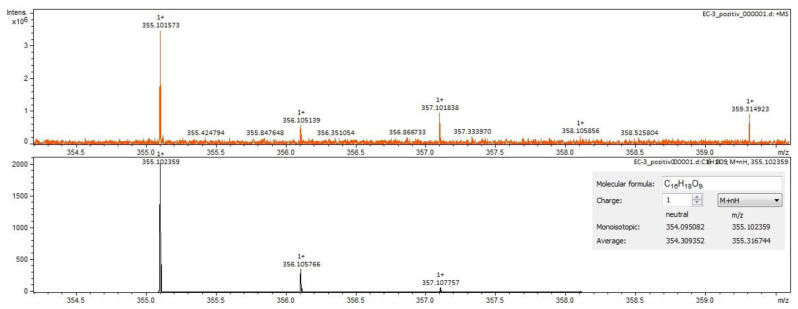
Chlorogenic acid identification in *Calendulae flos* extract–spectrogram (ESI+, water sample).

**Figure 5:**
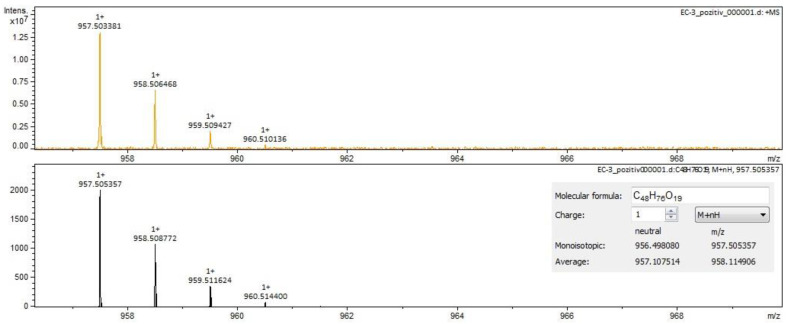
Calenduloside H identification in *Calendulae flos* extract–spectrogram (ESI+, methanol sample).

**Figure 6:**
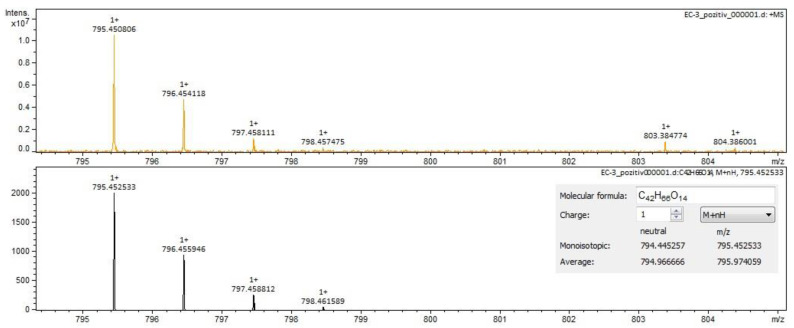
Calenduloside F/G identification in *Calendulae flos* extract–spectrogram (ESI+, methanol sample).

**Figure 7:**
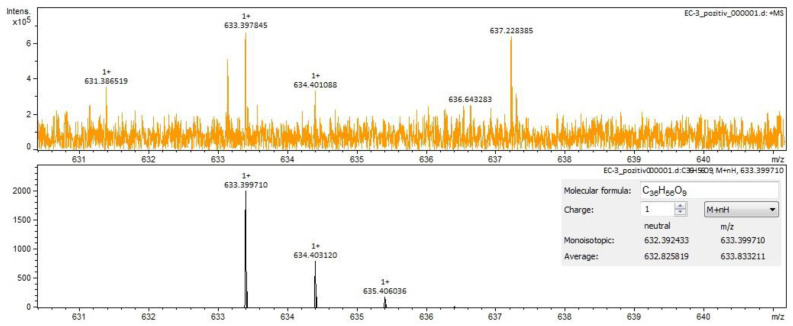
Calenduloside E identification in *Calendulae flos* extract–spectrogram (ESI+, methanol sample).

For *Calendulae flos* extract, rutin, narcissin, typhaneoside and chlorogenic acid (Figure 4) were identified by both ESI+ and ESI– in both solvents. Isoquercitrin was found by ESI+ and for ESI– only in the methanol sample. Isorhamnetin and quercetin were identified only by ESI+. Regarding the calendulosides, calenduloside H (Figure 5) was identified by ESI+ only in methanol sample and was identified by ESI– in both the methanol sample and water sample; calenduloside F/G (Figure 6) was identified by both types of ionization only in the methanol sample; calenduloside E (Figure 7) was identified only by ESI+ in the methanol sample.

### 2.3. Quantitative Spectrophotometric Analysis

The results of quantitative spectrophotometric analysis for three phytochemical classes are shown in Table 4, and boxplots for descriptive statistics are presented in Supplementary Materials Figures S29–S31.

The highest amount of phenolcarboxylic acids was found in *Sophorae flos* extract: 17.1399 g chlorogenic acid/100 g extract, 95% CI [16.5139; 17.7659]. Intermediate amounts are recorded for *Calendulae flos* extract: 3.8073 g chlorogenic acid/100 g extract, 95% CI [3.8638; 3.7509], and the lowest amount was found for *Ginkgo bilobae folium* extract: 2.1722 g chlorogenic acid/100 g extract, 95% CI [2.1007; 2.2437].

Regarding flavonoids, *Sophorae flos* extract stands out with the highest amount of flavones: 37.4473 g rutin/100 g extract, 95% CI [37.1959; 37.6987], being well known as a source for rutin extraction. It is followed by *Ginko bilobae folium* extract with a content of 2.3765 g rutin/100 g extract, 95% CI [2.3108; 2.4421]. The lowest content was found in *Calendulae flos* extract: 2.0129 g rutin/100 g extract, 95% CI [1.9924; 2.0334].

The highest content of polyphenols was recorded for *Sophorae flos* extract: 28.4903 g tannic acid/100 g extract, 95% CI [28.2607; 28.7198]. Intermediate amounts were found for *Ginkgo bilobae folium* extract: 7.2344 g tannic acid/100 g extract, 95% CI [7.1773; 7.2915]. *Calendulae flos* extract recorded the lowest content: 4.9029 g tannic acid/100 g extract, 95% CI [4.8385; 4.9672].

### 2.4. UHPLC-HRMS/MS Analysis

General screening of the extracts was performed for 152 compounds present in plant matrices (Supplementary Materials Table S1) belonging to different phytochemical classes. More than 30 compounds were found in each analyzed extract, of which 18 are common (five carboxylic acids and 13 flavonoids): quinic acid, azelaic acid, p-coumaric acid, syringic acid, caffeoylshikimic acid, quercetin, rutin, apigenin, naringenin, hyperoside, kaempferol-*O*-hexoside, chryosoeriol-7-hexoside, cynaroside, apigenin-7-*O*-glucosylhexoside, hispidulin-*O*-hexoside, isoquercitrin, isorhamnetin-3-*O*-hexoside and robinin.

**Figure 8:**
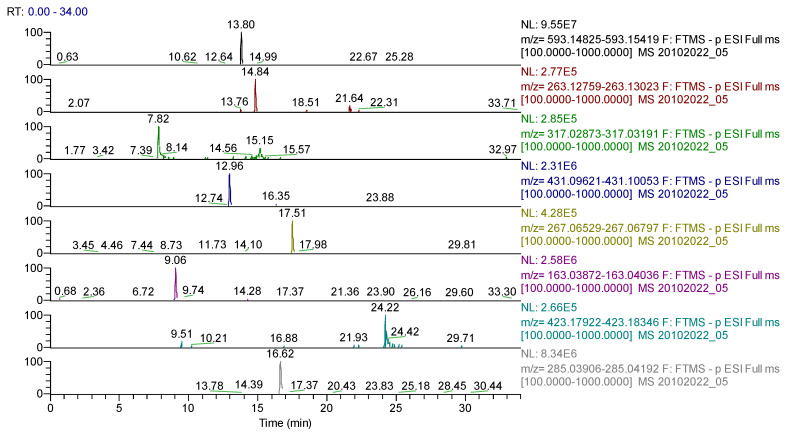
UHPLC–HRMS/MS chromatogram for *Sophorae flos* extract–identification of: apigenin-7-*O*–glucosylhexoside (*m*/*z* = 593.15122, Rt = 13.8); abscisic acid (*m*/*z* = 263.12891, Rt = 14.84); quercetagetin (*m*/*z*= 317.03032, Rt = 7.82); genistin (*m*/*z* = 431.09837, Rt = 12.96); formononetin (*m*/*z* = 267.06631, Rt = 17.51); p-coumaric acid (*m*/*z* = 163.03954, Rt = 9.06); sophoraflavone G (*m*/*z* = 423.18134, Rt = 24.22); kaempherol (*m*/*z* = 285.04049, Rt = 16.62).

For *Sophorae flos* extract, 43 compounds were identified (Table 5, Figure 8) belonging to the following phytochemical classes: 35 flavonoids, four phenolcarboxylic acids, one dicarboxylic acid, one cyclohexanecarboxylic acid, one sesquiterpene and one triterpene.

**Figure 9:**
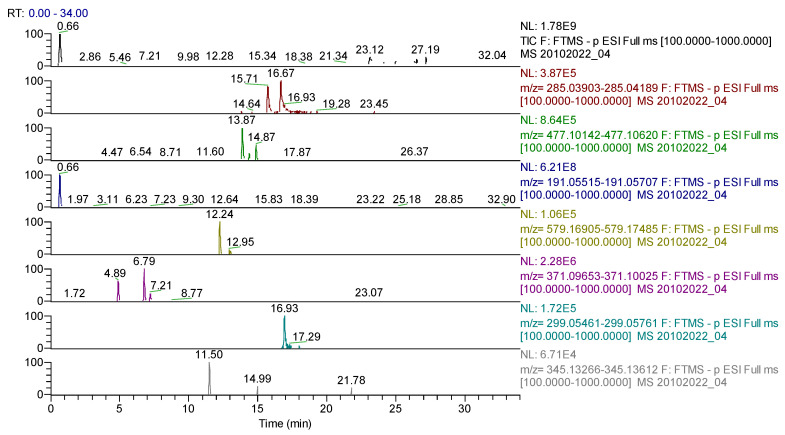
UHPLC–HRMS/MS chromatogram for *Ginkgo bilobae folium* extract– identification of: kaempherol (*m*/*z* = 285.04049, Rt = 16.67); baptigenin (*m*/*z* = 285.04046, Rt = 15.71); isorhamnetin 3-*O*-hexoside (*m*/*z* = 477.10387, Rt = 13.87); acid quinic (*m*/*z* = 191.05611, Rt = 0.66); narirutin (*m*/*z* = 579.17195, Rt = 12.24); naringin (*m*/*z* = 579.17185, Rt = 12.95); hidroxyferulic acid (*m*/*z* = 371.09839, Rt = 6.79); diosmetin (*m*/*z* = 299.05611, Rt = 16.93); cinaropicrin (*m*/*z* = 345.13439, Rt = 11.5).

For *Ginkgo bilobae folium* extract, 42 compounds were found (Table 6, Figure 9), classified as follows: 32 flavonoids, seven phenolcarboxylic acids, one dicarboxylic acid, one cyclohexanecarboxylic acid and one sesquiterpene.

**Figure 10:**
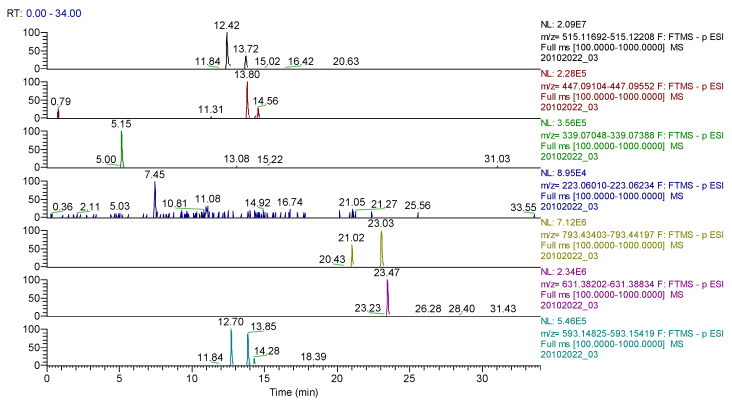
UHPLC–HRMS/MS chromatogram for *Calendulae flos* extract–identification of: cynarin (*m*/*z*= 515.11950, Rt = 12.42); quercitrin/isoquercitrin (*m*/*z* = 447.09331, Rt = 13.8); cichorin (*m*/*z* = 339.07218, Rt = 5.15); sinapic acid (*m*/*z* = 223.06122, Rt = 7.45); calenduloside G/F (*m*/*z* = 793.43800, Rt = 21.02/23.03); calenduloside E (*m*/*z* = 631.38518 Rt = 23.47); kaempherol-3-*O*-rutinoside (*m*/*z* = 593.15122, Rt = 12.7); apigenin-7-*O*–glucosylhexoside (*m*/*z* = 593.15122, Rt = 13.85).

For *Calendulae flos* extract, 35 compounds were identified (Table 7, Figure 10) belonging to several classes as follows: 17 flavonoids, nine phenolcarboxylic acids, two coumarins, two sesquiterpenes, two triterpenes, one diterpene, one dicarboxylic acid and one acid cyclohexanecarboxylic.

In all the analyzed extracts, the preponderance of flavonoids is noticeable.

Flavonoid compounds and phenolcarboxylic acids were the main classes determined using quantitative UHPLC-HRMS/MS method. The results are shown in Table 8 and are summarized as: 18 compounds (four phenolcarboxylic acids, 13 flavonoids and one sesquiterpene) for *Sophorae flos* extract, 16 compounds (five phenolcarboxylic acids, 10 flavonoids and one sesquiterpene) for *Ginkgo bilobae folium* extract and 11 compounds (six phenolcarboxylic acids, four flavonoids and one sesquitepene) for *Calendulae flos* extract. *Sophorae flos* extract had high contents in rutin (104,186.77 μg/g extract), isorhamnetin (97,049.32 μg/g extract) and quercetin (46,678.34 μg/g extract). In the *Ginkgo bilobae folium* extract, high amounts of isorhamnetin (5032.60 μg/g extract), quercetin (4504.66 μg/g extract) and rutin (3907.47 μg/g extract) were observed. In *Calendulae flos* extract, the highest amounts are represented by chlorogenic acid (20,676.63 μg/g extract), isorhamnetin (11,286.93 μg/g extract) and rutin (2165.42 μg/g extract).

Among all compounds determined quantitatively, eight were quantified in all three extracts: p-coumaric acid (SE > GE > CE), syringic acid (SE > CE > GE), gallic acid (SE > GE > CE), hyperoside (SE > GE > CE), rutin (SE > GE > CE), quercetin (SE > GE > CE), isorhamnetin (SE > CE > GE) and abscinic acid (SE > GE > CE). For the total polyphenolic content quantified by the UHPLC-HRMS/MS method, *Sophorae flos* extract (277.05 mg/g) was remarked, followed by *Calendulae flos* extract (36.39 mg/g) and then *Ginkgo bilobae folium* extract (19.32 mg/g).

### 2.5. Antioxidant Activity and Correlation Analysis

The antioxidant activity was determined by three in vitro methods: FRAP, DPPH and ABTS. The regression curves and their equations can be found in Supplementary Materials Figures S32–S34. Regarding ferric reduction assay, the best results were obtained for *Sophorae flos* extract (EC_50_ = 129.38 μg/mL), followed by *Calendulae flos* extract (EC_50_ = 437.00 μg/mL) and by *Ginkgo bilobae folium* extract (EC_50_ = 480.55 μg/mL). Against the DPPH radical, the best inhibition belonged to *Sophorae flos* extract (IC_50_ = 94.78 μg/mL), and the lowest inhibition was noticed for *Calendulae flos* extract (IC_50_ = 806.79 μg/mL). *Ginkgo bilobae folium* extract (IC_50_ = 518.49 μg/mL) had intermediate inhibitory activity. For the inhibition of the ABTS radical, the best results are also observed for *Sophorae flos* extract (IC_50_ = 71.51 μg/mL), followed by *Ginkgo bilobae folium* extract (IC_50_ = 193.89 μg/mL). The lowest inhibitory activity against ABTS radical was observed for *Calendulae flos* extract (IC_50_ = 369.34 μg/mL).

The correlation between the concentration of active compounds determined by spectrophotometric methods and the UHPLC method was evaluated, and the correlation between the results of all quantitative determinations (phenolcarboxylic acids, flavones and polyphenols) and the antioxidant activity by the three methods (FRAP, DPPH and ABTS) was also evaluated (Table 9, Table 10 and Table 11). Correlations and *p*-value results can be found in Supplementary Materials Tables S2–S5.

There is a positive Pearson correlation between the spectrophotometric methods and the UHPLC method (r > 0). A very strong correlation (r > 0.9) was observed between the amount of active principles determined by UHPLC and the results of spectrophotometric determinations for the content of phenolcarboxylic acids, flavones and polyphenols.

Regarding flavones content and polyphenolic content, a very strong correlation, appreciated by the absolute value of Pearson coefficient (│r│), was noted (│r│ > 0.9) for all methods measuring antioxidant activity. For phenolcarboxylic acids, a very strong correlation was observed for the values obtained by FRAP and DPPH methods (│r│ > 0.9), and a strong correlation (0.7 ≤ │r│ ≤ 0.89) was observed for the values obtained by ABTS method. The results for the correlation between the values obtained by quantitative UHPLC are presented as follows: very strong correlation for FRAP and DPPH (│r│ > 0.9) and strong correlation for ABTS (0.7 ≤ │r│ ≤ 0.89).

### 2.6. Molecular Docking Simulations

A molecular docking study was performed to investigate the potential of 25 identified phytochemicals to inhibit four matrix metalloproteinase subtypes (MMP-1, MMP-2, MMP-3 and MMP-9). Firstly, the docking procedure was validated by docking the co-crystallized ligands into the binding site, followed by the superposition of the predicted conformations on the experimentally determined coordinates in order to calculate the root mean square deviation (RMSD) values. The best RMSD values were obtained after docking the MMP-9 inhibitor (0.1552 Å), followed by MMP-3 (0.2375 Å), MMP-2 (0.3269 Å) and MMP-1 (0.4213 Å) inhibitors (Supplementary Materials Figure S35). The higher RMSD observed for the MMP-1 inhibitor can be explained by the differences in the orientation of the benzyl moiety, which, however, should have a minimum impact on predicting the interactions with the catalytic zinc. The redocked inhibitors were also considered positive controls. The redocking procedure yielded a binding energy of −6.467 kcal/mol for the MMP-1 inhibitor, −9.306 kcal/mol for the MMP-2 inhibitor, −7.509 kcal/mol for the MMP-3 inhibitor and −7.509 kcal/mol for the MMP-9 ligand, respectively. The ligand efficiency (LE) values were 0.2587, 0.2327, 0.3576 and 0.3142 (Supplementary Materials Table S6).

After screening the selected compounds on MMP-1, the estimated binding energies ranged between −9.47 and −6.076 kcal/mol (−8.178 ± 0.901 kcal/mol), and 23 compounds had binding energies lower than the positive control. In the case of MMP-2, the binding energies varied between −10.655 and −7.183 (−8.487 ± 0.919 kcal/mol), with six phytochemicals showing higher predicted affinities than the control. For MMP-3, the docking scores ranged from −9.547 to −6.522 kcal/mol (−8.247 ± 0.798 kcal/mol), and 21 compounds had higher scores when compared to the co-crystalized ligand. Lastly, the binding energies after docking with MMP-9 were estimated between −11.492 and −5.844 kcal/mol (−8.455 ± 1.220 kcal/mol), with 23 compounds showing binding energies lower than the known inhibitor. 

The highest predicted affinities after simulating interactions with the MMP-1 was obtained for rhamnetin, which, however, did not for any direct interactions with the catalytic zinc. Interestingly, sophoricoside had the highest predicted affinity for the other three MMPs (MMP-1, MMP-2 and MMP-3), although the phytochemical formed metal bonds only with the zinc atom bound to the MMP-9 catalytic site. The highest-ranking compounds that also interacted with the zinc atom within the binding site were narcissin for MMP-1, calenduloside G for MMP-2 and typhaneoside for MMP-3.

**Figure 11:**
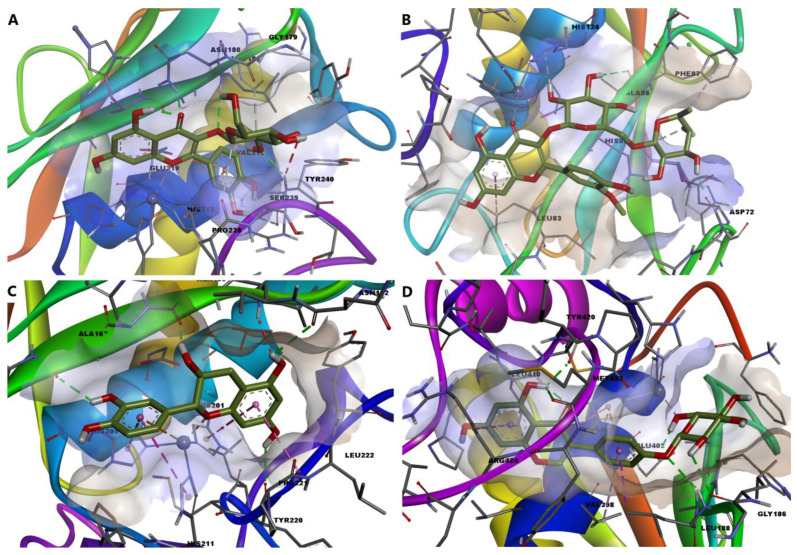
3D illustrations of the predicted protein-ligand complexes. (**A**)—predicted conformation of MMP-1-isoquercitrin complex; (**B**)—predicted conformation of MMP-2-narcissin complex; (**C**)—predicted conformation of MMP-3-catechin complex; (**D**)—predicted interaction of MMP-9-sophoricoside complex.

**Figure 12:**
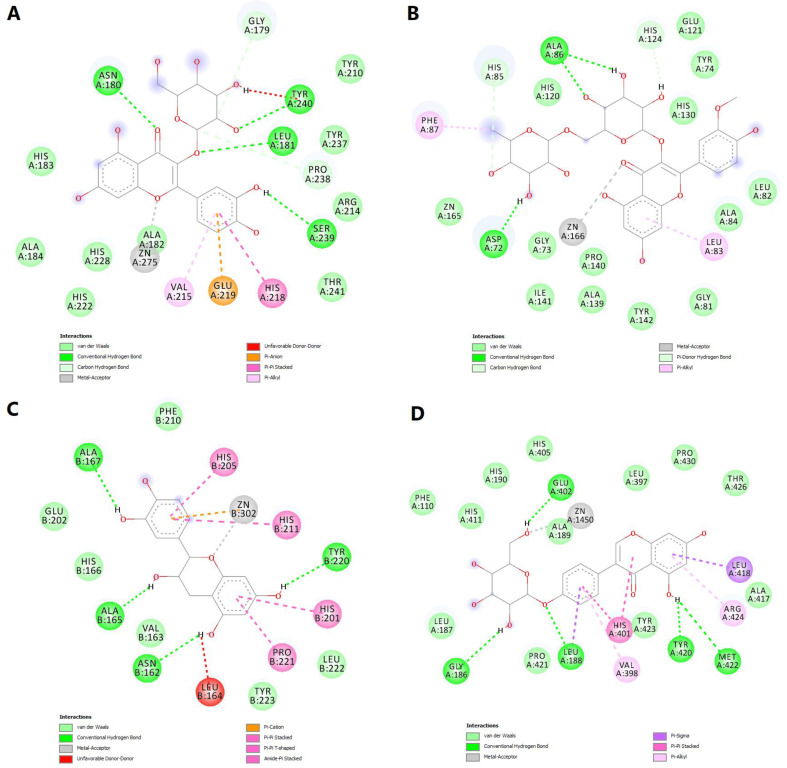
2D diagrams of the predicted molecular interactions between docked phytochemicals and MMPs active sites. (**A**)—predicted interactions between MMP-1 and isoquercitrin; (**B**)—predicted interactions between MMP-2 and narcissin; (**C**)—predicted interactions between MMP-3 and catechin; (**D**)—predicted interactions between MMP-9 and sophoricoside.

We further chose to discuss in more detail the predicted interactions between four phytochemicals and the active sites of the selected MMPs (Figure 11). Isoquercitrin had a binding energy of −8.529 kcal/mol for MMP-1. The ligand formed a metal bond with the catalytic zinc through one oxygen atom within the tetrahydroxyflavone substructure (Figure 12A). Four hydrogen bonds are formed with residues within the binding site, and the complex is further stabilized by nonpolar interactions such as pi-anion, pi-pi stacked, pi-alkyl and van der Waals interactions. However, an unfavorable donor–donor interaction was formed between the glucopyranosyl substructure and Tyr240.

Narcissin showed a binding energy of −7.919 kcal/mol for MMP-2 isoform. Following the docking simulation, a metal bond can be observed between the ketone moiety and the zinc atom. Moreover, three conventional hydrogen bonds are formed with Ala86 and Asp72, while carbon–hydrogen and pi-donor hydrogen bonds are formed with histidine residues 85 and 130 (Figure 12B).

Satisfying molecular interactions were predicted between catechin and MMP-3, with a binding energy of −7.864. Catechin binds the catalytic zinc through a metal bond and a pi–cation interaction. The flavonoid also formed four hydrogen bonds, several pi–pi stacked, pi–pi T-shaped and amide–pi stacked interactions, and an unfavorable donor–donor interaction between a hydroxyl group and Leu164 (Figure 12C).

Lastly, the isoflavone sophoricoside exhibited a binding energy of −11.492 kcal/mol for MMP-9, with a high ligand efficiency value (0.3707). Unlike the other three phytochemicals, sophoricoside interacted with the zinc atom through a hydroxyl group within the glycone substructure. The same hydroxyl moiety formed a hydrogen bond with Glu402, preventing the interaction between the key residue and the catalytic zinc. Four more hydrogen bonds are formed between the enzyme and other hydroxyl groups and the oxygen involved in the glycosidic bond. Hydrophobic interactions are also formed, such as pi–sigma, pi–pi stacked, pi–alkyl and van der Waals interactions (Figure 12D).

## 3. Discussion

Starting from plant sources known for their benefits in the treatment of vascular diseases (*Sophorae flos*, *Ginkgo bilobae folium* and *Calendulae flos*), extracts as dry powders were obtained. The extracts were preliminarily characterized regarding the content of active compounds and the antioxidant action. Qualitative (FT-ICR-MS, UHPLC-HRMS/MS) and quantitative (spectrophotometric, UHPLC-HRMS/MS) determinations were carried out, and the antioxidant activity was evaluated in vitro by three methods (FRAP, DPPH and ABTS).

Analysis by FT-ICR-MS identified common compounds (rutin, quercetin and isoquercitrin) and specific compounds (sophoricoside, ginkgolides and calendulosides). The compounds were mainly identified by ESI+; some of them were also identified by ESI– but less commonly. Regarding the solvents used for this method, most phytochemicals were identified in both methanolic and aqueous samples; some compounds were identified only in methanolic ones (e.g., kaikasaponins, calendulosides E, F/G, bilobetin).

Previous studies reported in the scientific literature revealed that sophoricoside isolated from *Sophora japonica* has anti-inflammatory effects; by selectively inhibiting COX-2, it also inhibits IL-6 (without having a cytotoxic effect), but not IL-1β and TNF-α. Even if the inhibitory effect was weak on GM-CSF (granulocyte-macrophage colony-stimulation factor), sophoricoside reduced inflammation by this pathway as well [27,28,29].

For *Ginkgo bilobae folium* extract, ginkgolides A, B and C were identified. Ginkgolide A and ginkgolide B are known to be involved in reducing the inflammatory response [30]. Ginkgolides B and C have been noted as antagonists of the platelet aggregation factor (PAF) receptor [31]. Bilobalide was also identified in the obtained extract, which was shown to contribute to the endothelial anti-inflammatory activity. Endothelial cells are activated by hypoxia (hypoxic status is characteristic of venous insufficiency). The ATP level decreases, and cytoplasmic Ca^2+^ increases. The inflammatory response occurs via the phospholipase A2 pathway; platelets are activated; and neutrophils adhere to the endothelial wall. Bilobalide delays the initiation of this cascade by preventing ATP depletion at the endothelial level (effect observed on HUVEC cells) [32].

Another important compound identified by FT-ICR-MS in *Ginkgo bilobae folium* extract is bilobetin. Studies on the anti-inflammatory effect were carried out finding that bilobetin significantly reduced the level of NO, TNF-α, IL-6 and PGE2 in RAW264.7 cells stimulated with lipopolysaccharides. The underlying mechanism is the reduction of mRNA levels for iNOS and COX-2 by bilobetin [30,33]. It has been reported that the bioflavonoids from *Ginkgo*, including bilobetin, have strong inhibitory effect on human thrombin, and flavonoids (kaempferol, apigenin, luteolin) have a moderate inhibitory effect. Human thrombin has two binding sites: exosite I (catalytic subunit, fibrinogen binding) and exosite II (heparin binding). Biflavonoids were found to bind to exosite I. Thus, *Ginkgo bilobae folium* extract acts bidirectionally to counteract the overexpressed prothrombotic status in chronic venous disease, inhibiting platelet aggregation (effect imprinted by the content of ginkgolides) and inhibiting thrombin (through the content of biflavonoids and flavones) [31,33].

In *Calendulae flos* extract, calendulosides (E,F/G,H) were identified by FT-ICR-MS. Studies revealed the antiapoptotic effect of calenduloside E on endothelial cells (HUVEC), targeting the modulation of Hsp90 (heat shock protein 90, expressed under stress factors and involved in the repair process) and the inhibition of caspase-3 [34,35,36].

From a quantitative point of view, extracts were analyzed for the content of flavones, phenolcarboxylic acids and total polyphenols, being the groups of active phytochemical compounds with a noteworthy role. The amount of flavones varied in the order: SE > GE > CE. For phenolcarboxylic acids, the recorded sequence was SE > CE > GE, and for polyphenols the recorded sequence was SE > GE > CE. Amounts depends on the vegetal source of the extract. Following these determinations, *Sophorae flos* extract was distinguished for the highest amounts of active compounds.

Through UHPLC-HRMS/MS analysis, the general screening of the extracts was carried out for the identification of polyphenolic compounds. More than 30 compounds were found in each extract. Most compounds belong to the flavonoid class, which are known for their vascular protective role. Flavonoids inhibit the inflammatory process (source of free radicals in chronic damage), decrease the activity of cyclooxygenases (COX-1, COX-2), lipoxygenase (LOX) and iNOS and decrease the production of proinflammatory cytokines (IL-6, IL-1β), TNF-α, chemokines, IL-8 and PGE2. They also inhibit the NF-kB pathway involved in gene transcription. Flavonoids exert their well-known antioxidant effect by inhibiting xanthine oxidase (promoting the formation of superoxide radical), lowering the activity of NADPH oxidase, neutralizing free radicals such as reactive oxygen species and stimulating the activity of endogenous antioxidant systems (superoxide dismutase, glutathione peroxidase). Thus, flavonoids prevent the propagation of inflammation and oxidative stress, reducing vascular damage [14,37].

Quantitative analysis performed by UHPLC-HRMS/MS method revealed high amounts of rutin, isorhamnetin and quercetin for *Sophorae flos* extract, revealed isorhamnetin, quercetin and rutin for *Ginkgo bilobae folium* extract and revealed chlorogenic acid, isorhamnetin and rutin for *Calendulae flos* extract. Their presence in extracts in a significant amount leads to the multimodal effect on the pathogenesis of chronic venous disease. Rutin has an important role in preventing endothelial dysfunction through the combination of three general effects: antioxidant, anti-inflammatory and antithrombotic. It exerts its antioxidant action through oxidative transformation into quinone that up-regulates the endogenous antioxidant response via the Nrf2 pathway. Endothelial damage is also prevented by promoting the release of vasodilator mediators (nitric oxide, endothelium-derived hyperpolarizing factor) and inhibiting the release of proangiogenic factors (VEGF and MMP-2). For the antithrombotic effect, it has been found to inhibit fibrin clot formation, decrease thrombin activity and suppress the interaction between thrombin and fibrinogen in both mice and humans. It additionally prolongs aPTT (intrinsic) and PT (extrinsic), inhibits platelet aggregation and prevents interactions between coagulation factors and platelet receptors [14,38,39,40]. Isorhamnetin has a complex mechanism to counteract endothelial damage; it acts mainly through the anti-inflammatory mechanism complemented by the anti-apoptotic action; it blocks signaling on the NF-kB pathway, decreases the level of VCAM-1, ICAM-1 and E-selectin (induced by TNF-α) and up-regulates the level the apoptotic protein AP-1 [41]. Quercetin also has multiple implications: it inhibits inflammatory mediators (IL, PG, NO, TNF-α), reduces the oxidative level and prevents apoptosis by inhibiting caspase-3. Another important aspect is tissue regeneration promoted by quercetin through the formation of collagen fibers and the production of binding substances to restore the skin; this effect would be beneficial for advanced stages of chronic venous disease with ulceration [37,42,43]. At the endothelial level, quercetin and chlorogenic acid have an antioxidant effect by reducing the level of xanthine oxidase 1 and increasing the level of superoxide dismutase and heme oxygenase 1. These two polyphenols also reduce the expression of ICAM-1, VCAM-1 and MCP-1 [44]. The endothelial protective activity of chlorogenic acid is based on the SIRT1/AMPK/PGC-1 pathway. It prevents AMPK phosphorylation, SIRT1 activation and the triggering of apoptosis. The antiapoptotic effect of chlorogenic acid is also supported by up-regulation of Bcl-2 expression (antiapoptotic) and down-regulation of Bax expression (pro-apoptotic, overexpressed under oxidative stress) [45,46].

The antioxidant activity of the obtained extracts was analyzed by three in vitro methods (FRAP, DPPH and ABTS). The prooxidant status is a source of pathogenesis in chronic venous disease, and the antioxidant action is one of the most important properties of natural compounds. Polyphenols neutralize radicals through two general steps: chain-breaking and single-electron transfer [47]. The action of venoactives is directed to the complexes of the respiratory chain that generate reactive species. One of their roles in venous insufficiency is to reduce ROS formed in the hypoxic conditions given by the pathological context [14].

*Sophorae flos* extract was noted for its antioxidant capacity highlighted by all three methods considering that, compared to the other two extracts, it showed the lowest concentration necessary for inhibiting the oxidative process. At the same time, it was also noted for the highest content of active principles both by spectrophotometric methods and by quantitative UHPLC. An important amount of polyphenols ensures a distinguished antioxidant action. Thus, the correlation between the results of spectrophotometric determinations and the UHPLC method was evaluated, as well as between the antiradical activity and the content of active principles in the extracts.

There is a very strong correlation between the results obtained by UHPLC and spectrophotometric determinations (r > 0.9). A correlation with statistical significance was noted between UHPLC and the content of phenolcarboxylic acids determined by spectrophotometric methods (*p* = 0.026, *p* < 0.05) as well as between UHPLC and the content of flavones (*p* = 0.043, *p* < 0.05). Quantitative UHPLC determinations were carried out for polyphenolic compounds belonging mainly to these two classes of active substances.

Regarding the correlation between the content of active compounds and antioxidant methods, a negative value for the Pearson coefficient is observed for all situations. Being an inverse correlation, the value of the inhibitory concentration (IC_50_/EC_50_) is the lower as the extract contains a greater amount of active compounds, imprinting greater antioxidant power.

Among the applied methods, for the FRAP method a statistically significant correlation was observed with the values of the content of active principles determined by quantitative UHPLC (*p* = 0.004, *p* < 0.05), as well as with the determination of the content of phenolcarboxylic acids (*p* = 0.022, *p* < 0.05) and determination of flavones content (*p* = 0.047, *p* < 0.05).

Even though not all correlations evaluated were statistically significant, very strong and strong correlations were observed between these methods, which confirms the reliability for antioxidant activity obtained by different assays. Antioxidant mechanisms being so different, the use of several methods can provide an overall perspective on antioxidant activity.

Previous studies highlighted that the levels of MMPs are increased in varicose veins with hydrostatic pressure, oxidative stress, hypoxia and inflammation favoring these changes. Lipodermatosclerosis (preceding phase of venous ulcer) is characterized by an increase in mRNA and protein expression for MMP-1 and MMP-2. Even if in this phase the collagen bundles are dense, they become loose due to the continuous activity of MMP-1 and MMP-2. In venous ulcers fluids, MMP-1 and MMP-2 are strongly active. MMPs are also involved in re-epithelialization. MMP-1 (by degrading collagenous matrix) and MMP-2 (by degrading non-collagenous matrix) facilitate keratinocytes migration; their activity decreases as re-epithelialization takes place. Moreover, MMP-3 was shown to have higher expression levels in varicose veins, contributing to vein wall weakness via collagen III degradation. MMP-9 was also previously associated with multiple forms of peripheral vascular disease, such as atherosclerosis, critical limb ischemia, intimal hyperplasia and varicose veins [48]. Thus, MMP activity imbalance should be prevented, since they are important components during re-epithelialization, but their overexpression in cases of venous ulcers impairs healing [4,5,6,7,8].

Due to the attractiveness of targeting MMPs for treating chronic venous disease, we performed a molecular docking study to evaluate the potential of several phytochemicals identified in the assessed plant extracts to inhibit their endopeptidase activity. Following the simulations, we identified several compounds that may inhibit MMP-1, MMP-2, MMP-3 and MMP-9 by binding to the catalytic Zn^2+^. Among the 25 screened compounds, seven interacted with Zn^2+^ within the MPP-1 active site (calendoflavoside, calenduloside G, chlorogenic acid, isoquercitrin, kaikasaponin III, narcissin and sophoricoside). The same number of phytochemicals also bound the catalytic zinc in MMP-2 (calendoflavoside, calenduloside G and H, ginkgolide B, kaikasaponin III, narcissin and rutin). For MMP-3, the 10 phytochemicals that successfully bound Zn^2+^ were calenduloside E, F, G, catechin, ginkgolide C, isoquercitrin, kaikasaponin III, narcissin, rutin and typhaneoside. Lastly, 12 compounds interacted directly with the zinc within MMP-9 active site: calendoflavoside, calenduloside E, F, G, ginkgolide A, B, C, kaikasaponin I, narcissin, rutin, sophoricoside and typhaneoside. Interestingly, calenduloside G and narcissin bound the Zn^2+^ contained by all four MMPs. Previous studies showed that narcissin can decrease the activity of secretion of MMP-2 and MMP-9 after exposure to UVB irradiation [49]. Similarly, isoquercitrin decreased the expression of MMP-1 and MMP-9 post irradiation [50]. Other studies revealed that green tea catechins inhibit the activity and neutrophil release of MMP-9 [51]. On the other hand, no previous studies investigated the effects of sophoricoside on MMPs activity.

## 4. Materials and Methods

### 4.1. Preparation of Vegetal Extracts

Vegetal product *Sophorae flos* (under the name *HuaiHua*) was purchased online from a producer from China. *Ginkgo bilobae folium* and *Calendulae flos* were purchased from a producer from Romania. Starting from the mentioned plant sources, dry extracts were obtained by ethanol reflux, rotavapor concentration (Buchi, R-215) and lyophilization (Christ Alpha). The vegetal product:solvent ratio was 1:10. It was maintained at reflux for 30 min (electric heating source). For *Calendulae flos* and *Ginkgo bilobae folium*, 70% ethanol was used as the extraction solvent, and for *Sophorae flos*, 80% ethanol was used. A successive two-step extraction was applied, and the filtrates were reunited and concentrated on a rotary evaporator and lyophilized. The extracts were conditioned in a desiccator.

### 4.2. FT-ICR-MS (Fourier-Transform Ion–Cyclotron-Resonance High-Resolution Mass Spectrometry) Method

Analysis was performed with the FT-ICR-MS system type solariX-XR QqqFT-ICR HR (Bruker Daltronics) with a 15T superconducting magnet, for both negative and positive ionization [52]. Parameters for negative ionization: sample injection flow rate of 120 μL/h, capillary 4700 V, end plate offset −800 V and carrier gas pressure (N_2_) 2.8 bar at 200 °C, with a flow rate of 3 L/min; the mass range was 92–1500 amu. Parameters for positive ionization: injection flow rate 120 μL/h, capillary 4500 V, end plate offset −700 V and carrier gas pressure (N_2_) 2.2 bar at 180 °C, with flow rate 3.5 L/min and mass range 92–1800 amu.

The samples were prepared by dissolving the extracts in solvents: ultrapure water and methanol (Sigma-Aldrich, Taufkirchen, Germany). A quantity of 10 mg of extract dissolved in 10 mL solvent in conical tubes (Thermo Scientific, Dreieich, Germany) was shaken vigorously and kept at rest until the foam disappeared; then 100 μL was taken, adding 10 μL of formic acid and diluting to 10 mL. The sample thus obtained is injected into the system. Based on the molecular formula, ESI– (1M-nH) and ESI+ (1M + nH) ionization templates were generated (Bruker Compass Data Analysis) and identified in the obtained spectrograms.

### 4.3. Quantitative Spectrophotometric Analysis

#### 4.3.1. Phenolcarboxylic Acids Content

Phenolcarboxylic acids form nitrosoderivatives with Arnow’s reagent (10 g of sodium nitrite + 10 g of sodium molybdate dissolve in 100 mL of water), in acid medium (0.5 N hydrochloric acid), which spontaneously tautomerize to oximes which are red colored and are soluble in alkaline medium (sodium hydroxide 8.5%). The intensity of the coloration is directly proportional to the amount of phenolcarboxylic acids in the sample. The main reactive is nitric acid which is obtained in the reaction medium from sodium nitrite and hydrochloric acid.

For *Sophorae flos* extract: 0.15 g extract in a 25 mL volumetric flask, adjusted to mark with 80% ethanol, sample range from 0.1 mL to 0.6 mL, progression rate of 0.1 mL, 3 sets of determinations per flask, 6 determinations per set. For *Ginkgo bilobae folium* extract: 0.25 g of extract in a 25 mL volumetric flask, adjusted to the mark with 70% ethanol. Sample range from 0.5 mL to 2.5 mL, progression rate 0.4 mL. One set of determinations per flask with six determinations per set was performed. For *Calendulae flos* extract: 0.25 g of extract in a 25 mL volumetric flask, adjusted to mark with 70% ethanol, sample range from 0.3 mL to 1.3 mL, progression rate of 0.2 mL. Two sets of determinations were made for each flask and six determinations per set.

The samples were prepared from the analyte solution by taking different volumes (the sample range) in 10 mL volumetric flasks. Add 2 mL of 0.5 N hydrochloric acid, 2 mL of Arnow reagent, 2 mL of 8.5% sodium hydroxide and adjust to the mark with distilled water. Controls are prepared analogously but without the Arnow reagent. Absorbances were measured by spectrophotometer (Jasco V-530), against the controls at the wavelength λ = 510 nm. The results were obtained using a standard curve of chlorogenic acid obtained under the same experimental conditions (Supplementary Materials Figure S36). All reagents were purchased from Sigma-Aldrich.

#### 4.3.2. Flavonoid Content

In the presence of bivalent and trivalent metallic ions (aluminum chloride solution 2.5%), flavones form yellow colored chelates with yellow-greenish fluorescence stable in 10% sodium acetate medium. 

For *Sophorae flos* extract: 0.1 g extract in a 50 mL volumetric flask, adjusted to the mark with 80% ethanol, sample range from 0.1 mL to 0.4 mL, progression rate 0.05 mL, 3 sets of determinations per flask, 7 determinations per set. For *Ginkgo bilobae folium* extract: 0.15 g extract in a 25 mL volumetric flask, adjusted to the mark with 70% ethanol, sample range from 0.4 mL to 2 mL, progression rate 0.2 mL, one set of determinations per flask, 9 determinations per set. For *Calendulae flos* extract: 0.25 g of extract in 25 mL volumetric flask, adjusted to the mark with 70% ethanol, sample range from 0.4 mL to 1.5 mL, progression rate of 0.1 mL, one set of determinations per flask, 12 determinations per set.

Samples were prepared by taking successive volumes in 10 mL volumetric flasks. Add 2 mL of 10% sodium acetate, then add 1 mL of 2.5% aluminum chloride solution and adjust to the mark with the extraction solvent (ethanol 70% or ethanol 80%). In parallel with the samples, controls are prepared by diluting equivalent volumes with solvent in 10 mL flasks, without adding reagents. Allow to stand for 30–45 min and measure absorbances against controls at 427 nm (Jasco V-530). For results, a standard curve was used of rutin obtained under the same experimental conditions (Supplementary Materials Figure S37). All reagents were purchased from Sigma-Aldrich.

#### 4.3.3. Polyphenolic Content

Polyphenols are oxidized by Folin–Ciocâlteu reagent (molybdotungstate (Na_2_WO_4_/Na_2_MoO_4_)) when the Mo^4+^ ion is the result, which imprints a blue color in sodium carbonate solution at the limit of solubility (200 g/L). Diluted Folin–Ciocâlteu reagent is prepared by dilution of the concentrate with distilled water (1:1). 

For *Sophorae flos* extract: 0.1 g extract in a 100 mL volumetric flask, adjusted to mark with 80% ethanol, sample range from 0.1 mL to 0.3 mL, progression rate 0.025 mL, 3 sets of determinations per flask, 9 determinations per set. For *Ginkgo bilobae folium* extract: 0.15 g extract in 100 mL volumetric flask, adjusted to mark with 70% ethanol, sample range from 0.2 mL to 0.45 mL, progression rate 0.05 mL, 3 sets of determinations per flask, 7 determinations per set. For *Calendulae flos* extract: 0.15 g extract in a 50 mL volumetric flask, adjusted to mark with 70% ethanol, sample range from 0.15 mL to 0.45 mL, progression rate 0.05 mL, 3 sets of determinations per flask, 7 determinations per set.

Samples were prepared by taking progressively increasing volumes (up to a maximum of 0.9 mL) into volumetric flasks of 10 mL. Add distilled water up to 1 mL, then add 1 mL diluted Folin–Ciocâlteu reagent and adjust to the mark with the sodium carbonate solution (200 g/L). The absorbances were determined against control prepared from 1 mL of distilled water and 1 mL of Folin–Ciocâlteu reagent, adjusted to the mark with sodium carbonate. The samples are kept in the dark for 40 min; then, absorbances were measured by spectrophotometer (Jasco V-530) at a wavelength of 725 nm. The results were calculated using standard curve of tannic acid obtained under the same experimental conditions (Supplementary Materials Figure S38). All reagents were purchased from Sigma-Aldrich.

### 4.4. UHPLC-HRMS/MS Analysis

Materials used: reference standards (Sigma-Aldrich, Germany), methanol, ethanol, formic acid and ultrapure water (Merck, București, Romania) and Pierce LTQ Velos ESI calibration solutions for positive and negative ions consisting of mixtures of purified ionizable molecules (Thermo Fisher Scientific, Dreieich, Germany). Methanolic stock solutions (1 mg/mL) were prepared for each compound. Successive dilutions were made (range 0.05–1 μg/mL) and were stored at −20 °C until use.

The chromatographic system used (Thermo Scientific Dionex Ultimate 3000 UHPLC) consists of: pump (Series RS), column compartment (Series TCC-3000RS), automatic injector (Series WPS-3000RS) and Chromeleon 7.2 Software (Thermo Fisher Scientific). The stationary phase is represented by Accucore UHPLC Column C18 (150 × 2.1 mm, 2.6 μm) (Thermo Fisher Scientific). The mobile phase has two components: eluent A (ultrapure water with 500 μL/L formic acid) and eluent B (methanol with 500 μL/L formic acid). Gradient elution was applied as follows: 0–1 min 100% A, 1–10 min linear increase to 30% B, 10–26 min linear increase to 100% B, hold 4 min, 30–32.5 min decrease to 0% B and equilibration time of 2.5 min. Working conditions: mobile phase flow 0.3 mL/min, 35 min and temperature 40 °C [53].

Mass spectrometry system is based on spectrometer (Q-Exactive Mass Spectrometer) with electrospray ionization source (HESI-Heated ElectroSpray Ionization). Ionization source conditions: nitrogen flux for sheath gas was 8 units and for auxiliary gas was 6 units; temperature was 300 °C (source heater, capillary and auxiliary gas heater); voltage was 2800 V; S-lens RF level was 50. Resolving power for full screening was 70,000 FWHM *m*/*z* 200; screening range was *m*/*z* 100–1000 Da; AGC (Automatic Gain Control) was set to 3 × 10^6^; injection time was 200 ms, 2 scans/s. Structural information was obtained by the vDIA (variable Data Independent Acquisition). The scan events (six) were performed as follows: one event with previous mentioned parameters and five MS-MS events. In the second stage analysis (MS2), *m*/*z* variations were selected consecutively as follows: 95–205, 195–305, 295–405, 395–505 and 500–10,005 were fragmented by HCD (Higher-energy collisional dissociation) cells at 30, 60 and 80 NCE (Normalized Collision Energy) and measured in five separate Orbitrap scans at 35,000 FWHM resolving power. C-trap parameters for all scans: AGC target of 1 × 10^6^, injection time 100 ms. Data analysis was performed by Quan/Qual Browser Xcalibur 2.3 (Thermo Fisher Scientific) with the accepted mass error of 5 ppm [53].

The analysis consists in detection of at least two fragment ions by comparison with reference standards, and for compounds without a reference standard, structures were presumed by high-precision analysis of deprotonated precursors and fragment ions of specific components. Elemental chemical composition was determined (within 2 ppm mass error) using Chemspider database. Fragment ions from MS-MS analysis were used for structure confirmation by comparison with data from NORMAN MassBank, mzCloude Advanced Mass Spectral Database and PubChem. ACDLabs MS Fragmenter 2019.2.1 fragmentation template generator software was used.

### 4.5. In Vitro Antioxidant Activity

#### 4.5.1. Ferric Reducing Antioxidant Power Assay (FRAP)

The antioxidant analyte reacts with Fe^3+^ reducing to Fe^2+^ which imprints blue color. The intensity of the coloration is directly proportional to the antioxidant activity [52,53,54,55,56].

All reagents used were purchased from Sigma-Aldrich, Germany.

Analysis solution was prepared by dissolving the extract in the extraction solvent and were prepared successive dilutions. For *Sophorae flos* extract: 0.1 g extract in a 50 mL volumetric flask, adjusted to mark with 80% ethanol, progressive sample range from 0.1 mL to 1.4 mL, progression rate 0.1 mL. For *Ginkgo bilobae folium* extract and *Calendulae flos* extract: 0.1 g extract in a 50 mL volumetric flask, adjusted to mark with 70% ethanol, sampling range from 0.5 mL to 5 mL in progression at the rate of 0.5 mL.

The sample volumes were diluted in 10 mL volumetric flasks, adjusted to the mark with the solvent. A total of 2.5 mL from each volumetric flask was taken in test tubes, and 2.5 mL of phosphate buffer was added with pH = 6.6, 2.5 mL of 1% potassium hexacyanoferrate solution. The test tubes are kept in the water bath at 50 °C for 20 min. Add 2.5 mL of 10% trichloroacetic acid solution. A total of 2.5 mL of the obtained solution was taken in another test tube; 2.5 mL of distilled water and 0.5 mL of 0.1% ferric chloride solution were added. Kept at rest for 10 min, the absorbances were measured at λ = 700 nm (spectrophotometer Jasco V-530), compared to the control (prepared under the same conditions without sample solution). The EC_50_ value, which represents the sample concentration at which the absorbance has a value of 0.5 or half the concentration at which the antioxidant activity is maximum, was determined by the equation of the trendline (Supplementary Materials Figure S32).

#### 4.5.2. 2,2-Diphenyl-1-Picrylhydrazyl Free Radical Scavenging Assay (DPPH)

Under the action of an antioxidant, the purple free radical 2,2-diphenyl-1-picrylhydrazyl (DPPH) forms its corresponding yellow hydrazine [54,57].

All reagents used were purchased from Sigma-Aldrich, Germany.

DPPH solution was obtained from 0.0039 g DPPH dissolved in 100 mL absolute ethanol. The solution was prepared on the day of the determinations and kept in the dark; the absorbance of the solution was determined. The analyte solutions were prepared by dissolving extracts, and then dilutions are made in 10 mL volumetric flasks. Dilutions are obtained analogously to the protocol described for the FRAP method with the following changes: narrowing the sample range for *Sophorae flos* extract 0.1–0.8 mL. From each dilution, 0.5 mL was mixed with 3 mL of DPPH solution, and the samples rested for 30 min in the dark. The samples absorbances were measured against absolute ethanol by spectrophotometer (Jasco V-530), λ = 515 nm. The inhibition was calculated according to formula:

(1) $$I\%=\frac{A_{control}-A_{sample}}{A_{control}}\times 100$$

where *I* = inhibition of DPPH; *A_control_* = absorbance of DPPH solution without presence of antioxidant; *A_sample_* = absorbance of the sample with DPPH solution in presence of extract. The IC_50_ value was determined from inhibition curves and their linear equations (Supplementary Materials Figure S33).

#### 4.5.3. 2,20-Azinobis-3-Ethylbenzotiazoline-6-Sulfonic Acid Assay (ABTS)

The turquoise colored ABTS radical results from the reaction of a strong oxidizing agent (potassium persulfate) with the ammonium salt of 2,2’-azino-bis(3-ethylbenzothiazoline-6-sulfonic acid). Under the action of the antioxidant, the intensity of the color is reduced to colorless [55,57,58]. The ABTS radical solution is prepared by mixing two solutions: solution 1 (ammonium salt 7.4 mM) and solution 2 (potassium persulfate 2.6 mM). A total of 20 mL of solution 1 was added to 20 mL of solution 2 and kept in the dark for 16 h. A total of 1 mL of the mixture was diluted in a 50 mL volumetric flask, adjusted to mark with absolute ethanol, resulting the ABTS solution for reaction. The absorbance was determined by spectrophotometer (Jasco V-530), λ= 734 nm. All reagents used were purchased from Sigma-Aldrich, Germany.

Analyzed solutions are obtained from the extracts, and dilutions were obtained in 10 mL volumetric flasks. For *Sophorae flos* extract: 0.1 g extract in a 50 mL volumetric flask, adjusted to the mark with 80% ethanol, sample range from 0.1 mL to 0.6 mL, progression rate 0.05 mL. For *Ginkgo bilobae folium* extract: 0.1 g extract in a 50 mL volumetric flask, adjusted to the mark with 70% ethanol, sample range from 0.2 mL to 1.6 mL, progression rate of 0.2 mL. For *Calendulae flos* extract: 0.1 g of extract in a 50 mL volumetric flask, adjusted to mark with 70% ethanol, sample range from 0.2 mL to 2 mL, progression rate 0.2 mL. 0.5 mL of each dilution brought into vials, in contact with 3 mL of ABTS, kept in the dark for 5–6 min. Absorbances was measured against absolute ethanol.

The inhibition was calculated according to previously mentioned Formula (1), where *I* = inhibition of ABTS; *A_control_* = absorbance of ABTS solution without presence of antioxidant; *A_sample_* = absorbance of the sample with ABTS solution in presence of extract. The IC_50_ value was calculated from inhibition curves and their linear equations (Supplementary Materials Figure S34).

### 4.6. Molecular Docking

Molecular docking simulations were carried out for the phytoconstituents that were identified in the three plant extracts, to investigate their potential to inhibit activity of MMPs. Crystal structures of catalytic domains of target proteins in complex with known inhibitors were retrieved from RCSB PDB database: MMP-1 (PDB ID: 1HFC, 1.50 Å resolution [59]), MMP-2 (PDB ID: 1HOV [60]), MMP-3 (PDB ID: 4G9L, 1.88 Å resolution [61]) and MMP-9 (PDB ID: 1GKC, 2.30 Å resolution [62]). The protein structures were prepared for docking using YASARA Structure [63], by removal of water molecules, correction of structural errors, protonation according to the physiological pH (7.4), optimization of the hydrogen-bonding network and energy minimization using YASARA2 forcefield. The co-crystallized inhibitors were removed and redocked into the binding site for validation. The predicted conformation of the known inhibitors were superposed on the initial coordinates to calculate the root mean square deviation (RMSD) values [53].

The virtual library containing the identified phytochemicals was prepared by retrieving the SMILES codes from PubChem database. Thereafter, the 3D structures were generated using DataWarrior 5.2.1 [64], were energetically minimized using MMFF94s+ forcefield and were protonated according to the physiological pH. AutoDock Vina v1.1.2 docking algorithm [65] was used for the virtual screening, performing 12 runs for each compound. The docking results were retrieved as binding energy (ΔG, kcal/mol) and ligand efficiency (LE, ΔG\no. of heavy atoms) of the best binding pose. The molecular interactions predicted between the ligands and target proteins were analyzed using BIOVIA Discovery Studio Visualizer (BIOVIA, Discovery Studio Visualizer, Version 17.2.0, Dassault Systèmes, 2016, San Diego, CA, USA). The docked natural compounds were considered potential MMP inhibitors if they had good predicted affinities and interacted directly with the catalytic zinc.

### 4.7. Statistical Analysis

The determination of Pearson correlation was carried out using IBM SPSS Statistics Software Version v.28.0 (IBM Corporation, Chicago, IL, USA). The essential conditions for the application of statistical tests (such as the normality of data and the homogeneity of variances) were evaluated for each set of experimental data before running the correlation analysis. The normal distribution of the data on the symmetrical bell curve of Gauss was assessed by the Shapiro–Wilk test, by the Kolmogorov-Smirnov test and by histograms. The Levene’s test was applied to verify the homogeneity of variances for the experimental data sets. When certain experimental data deviate from the Gaussian distribution, they were transformed by the logarithm in base 10, so that it becomes normally distributed and can be subjected to parametric statistical tests. Interpretations about the Pearson correlation coefficient were made after the mandatory application criteria were met (continuity of variables, independence of measurements, normality and linearity of data and absence of outliers). The chosen level of significance was set at 0.05, and the results were considered significant when *p* < 0.05. Additionally, Μicrosoft Excel v.2019 was used for descriptive statistics and calculation of the confidence interval.

## 5. Conclusions

In the current research, three plant extracts from three different sources (*Sophorae flos*, *Ginkgo bilobae folium* and *Calendulae flos*) were obtained and preliminarily characterized. The phytochemical composition was determined qualitatively (FT-ICR-MS, UHPLC-HRMS/MS) and quantitatively (spectrophotometric methods and UHPLC-HRMS/MS). Chronic venous disease involves complex pathophysiological mechanisms summarized by favoring the prooxidant, proinflammatory and prothrombotic status. The active phytochemicals identified in the obtained extracts have multiple implications for counteract pathogenesis, and one of the most important effects is the antioxidant one. In this context, the antioxidant activity of the extracts was evaluated by three methods (FRAP, ABTS and DPPH) and proved to be strong correlated with the active compounds amount. Among the obtained extracts, *Sophorae flos* extract was noted both for the content of active phytochemicals (phenolcarboxylic acids, flavones, polyphenols) and its antioxidant activity. The other extracts (*Ginkgo bilobae folium*, *Calendulae flos*), although they have less amounts of active principles and low antioxidant action compared to *Sophorae flos* extract, are promising agents in the treatment of chronic venous disease through their phytochemical composition. Moreover, molecular docking simulations revealed the potential of several phytoconstituents to inhibit the catalytic activity of four MMP isoforms. Further research will aim cytotoxicity assays, activity on endothelial cell lines and including the extracts into a nanoformulation.

## Figures and Tables

**Table 1:** Identified compounds in *Sophorae flos* extract by FT-ICR-MS.

Compound	ESI+		ESI−	
	w	met	w	met
rutin	+	+	+	+
quercetin	+	+	+	+
isorhamnetin/rhamnetin	+	+	–	+
kaempferol/fisetin	+	+	–	+
isoquercitrin	+	+	–	+
sophoricoside	+	+	–	–
kaikasaponin I	–	+	–	+
kaikasaponin III	–	+	–	–

w = water sample; met = methanol sample.

**Table 2:** Identified compounds in *Ginkgo bilobae folium* extract by FT-ICR-MS.

Compound	ESI+		ESI–	
	w	met	w	met
rutin	+	+	+	+
bilobalid	+	+	+	+
quercetin	+	+	+	–
ginkgolide A	+	+	–	–
ginkgolide B	+	+	–	+
ginkgolide C	+	+	–	–
kaempferol	+	+	–	–
isoquercitrin	+	+	–	–
isorhamnetin/rhamnetin	+	+	–	–
catechin	+	+	–	–
bilobetin	–	+	–	–
caffeic acid	–	+	–	–

w = water sample; met = methanol sample.

**Table 3:** Identified compounds in *Calendulae flos* extract by FT-ICR-MS.

Compound	ESI+		ESI–	
	w	met	w	met
rutin	+	+	+	+
narcissin/calendoflavoside	+	+	+	+
typhaneoside	+	+	+	+
chlorogenic acid	+	+	+	+
isoquercitrin	+	+	–	+
isorhamnetin/rhamnetin	+	+	–	–
quercetin	+	+	–	–
calenduloside E	–	+	–	–
calenduloside F/G	–	+	–	+
calenduloside H	–	+	+	+

w = water sample; met = methanol sample.

**Table 4:** Quantitative spectrophotometric analysis.

Extract	Phenolcarboxylic Acids Content (g Chlorogenic Acid/100 g Extract)	Flavonoid Content (g Rutin/100 g Extract)	Polyphenolic Content (g Tannic Acid/100 g Extract)
SE	17.1399	37.4473	28.4903
	[16.5139; 17.7659] *	[37.1959; 37.6987] *	[28.2607; 28.7198] *
GE	2.1722 [2.1007; 2.2437] *	2.3765 [2.3108; 2.4421] *	7.2344 [7.1773; 7.2915] *
CE	3.8073 [3.8638; 3.7509] *	2.0129 [1.9924; 2.0334] *	4.9029 [4.8385; 4.9672] *

SE = *Sophorae flos* extract; GE = *Ginkgo bilobae folium* extract; CE = *Calendulae flos* extract; * Confidence interval for mean at confidence level 95%.

**Table 5:** Qualitative analysis of *Sophorae flos* extract by UHPLC-HRMS/MS.

Compound	Molecular Formula	Adduct Ion (*m*/*z*) Monitored Negative Ion	Retention Time (min)	Phytochemical Class
quinic acid	C_7_H_12_O_6_	191.05611	0.66	Cyclohexanecarboxylic acid
hidroxyferulic acid	C_16_H_20_O_10_	371.09839	6.77	phenolcarboxylic acids
caffeoylshikimic acid	C_10_H_15_O_9_	278.06435	7.65	phenolcarboxylic acids
myricetin	C_15_H_10_O_8_	317.03032	7.82	flavonoids
quercetagetin (6-hydroxyquercetin)	C_15_H_10_O_8_	317.03032	7.82	flavonoids
syringic acid	C_9_H_10_O_5_	197.04555	8.86	phenolcarboxylic acids
p-coumaric acid	C_9_H_8_O_3_	163.03954	9.06	phenolcarboxylic acids
hyperoside (quercetin-3-hexoside)	C_21_H_20_O_12_	463.08768	9.63	flavonoids
phlorizin	C_21_H_24_O_10_	435.12969	10.27	flavonoids
quercetin-3-*O*-rutinoside	C_33_H_40_O_21_	771.19896	10.37	flavonoids
isorhamnetin-3-*O*-hexoside	C_22_H_22_O_12_	477.10387	11.97/13.86	flavonoids
sissotrin(biochanin A7-*O*-β-D-hexoside)	C_22_H_22_O_10_	445.11404	12.5/15.91	flavonoids
rutin (quercetin-3-rutinoside)	C_27_H_30_O_16_	609.14613	12.58	flavonoids
robinin	C_33_H_40_O_19_	739.20913	12.67	flavonoids
genistin	C_21_H_20_O_10_	431.09837	12.96	flavonoids
naringin	C_27_H_32_O_14_	579.17185	13.00	flavonoids
chryosoeriol-7-hexoside	C_22_H_22_O_11_	461.10893	13.40	flavonoids
apigenin–7-rutinoside	C_27_H_30_O_14_	577.15630	13.49	flavonoids
hispidulin-*O*-hexoside/isomers	C_22_H_22_O_11_	461.10893	13.64	flavonoids
kaempferol (or luteolin)-*O*-hexoside/isomers	C_21_H_20_O_11_	447.09331	13.75	flavonoids
cynaroside (luteolin-7-hexoside)	C_21_H_20_O_11_	447.09328	13.75	flavonoids
isoquercitrin/quercitrin (quercetin-3-rhamnoside)	C_21_H_20_O_11_	447.09331	13.75	flavonoids
apigenin-7-*O*-glucosylhexoside	C_27_H_30_O_15_	593.15122	13.80	flavonoids
hispidulin-7-rutinoside/isomers	C_28_H_32_O_15_	607.16684	13.82	flavonoids
isorhamnetin-3-*O*-rutinoside	C_28_H_32_O_16_	623.16178	13.96	flavonoids
azelaic acid	C_9_H_16_O_4_	187.09761	14.10	dicarboxylic acids
vitexin 2-*O*-rhamnoside	C_27_H_30_O_14_	577.15630	14.29	flavonoids
abscisic acid	C_15_H_20_O_4_	263.12891	14.84	sesquiterpenes
quercetin	C_15_H_10_O_7_	301.03540	15.15	flavonoids
naringenin	C_15_H_12_O_5_	271.06122	15.63	flavonoids
hesperitin	C_16_H_14_O_6_	301.07179	15.71	flavonoids
gallocatechin/epigallocatechin	C_15_H_14_O_7_	305.06668	15.73	flavonoids
afrormosin	C_17_H_14_O_5_	297.07687	16.39	flavonoids
kaempferol	C_15_H_10_O_6_	285.04049	16.62	flavonoids
apigenin	C_15_H_10_O_5_	269.04502	16.83	flavonoids
6-methoxyluteolin	C_16_H_12_O_7_	315.05105	16.85	flavonoids
diosmetin	C_16_H_12_O_6_	299.05611	16.92	flavonoids
pseudobaptigenin	C_16_H_10_O_5_	281.04557	17.36	flavonoids
formononetin	C_16_H_12_O_4_	267.06631	17.51	flavonoids
glycitein	C_16_H_12_O_5_	283.06122	19.65	flavonoids
ginkgetin	C_32_H_22_O_10_	565.11404	21.37	flavonoids
calenduloside G/isomers	C_42_H_66_O_14_	793.43800	22.99	triterpenes
sophoraflavanone G/isomers	C_25_H_28_O_6_	423.18134	24.22	flavonoids

**Table 6:** Qualitative analysis of *Ginkgo bilobae folium* extract by UHPLC-HRMS/MS.

Compound	Molecular Formula	Adduct Ion (*m*/*z*) Monitored Negative Ion	Retention Time (min)	Phytochemical Class
quinic acid	C_7_H_12_O_6_	191.05611	0.66	cyclohexanecarboxylic acids
daphnin	C_15_H_16_O_9_	339.072155	5.18	flavonoids
gallocatechin/epigallocatechin	C_15_H_14_O_7_	305.06668	5.47	flavonoids
isorhamnetin-3-*O*-hexoside	C_22_H_22_O_12_	477.10381	5.69	flavonoids
chlorogenic acid	C_16_H_18_O_9_	353.08783	6.23	phenolcarboxylic acids
hidroxyferulic acid	C_16_H_20_O_10_	371.09839	6.79	phenolcarboxylic acids
caffeoylshikimic acid	C_10_H_15_O_9_	278.06435	7.70	phenolcarboxylic acids
epicatechin	C_15_H_14_O_6_	289.07176	8.01	flavonoids
syringic acid	C_9_H_10_O_5_	197.04555	8.86	phenolcarboxylic acids
p-coumaric acid	C_9_H_8_O_3_	163.03954	9.09	phenolcarboxylic acids
hyperoside (quercetin-3-hexoside)	C_21_H_20_O_12_	463.08768	9.64	flavonoids
quercetin-3-*O*-rutinoside	C_33_H_40_O_21_	771.19896	10.25	flavonoids
cynaropicrin	C_19_H_22_O_6_	345.13439	11.50	sesquiterpenes
narirutin (naringenin-7-*O*-rutinoside)	C_27_H_32_O_14_	579.17195	12.24	flavonoids
cynarin (1,5-di-*O*-caffeoylquinic acid)	C_25_H_24_O_12_	515.11950	12.37	phenolcarboxylic acids
robinin	C_33_H_40_O_19_	739.20913	12.39	flavonoids
rutin (quercetin-3-rutinoside)	C_27_H_30_O_16_	609.14613	12.60	flavonoids
naringin	C_27_H_32_O_14_	579.17185	12.93	flavonoids
chryosoeriol-7-hexoside	C_22_H_22_O_11_	461.10893	13.33/14.96	flavonoids
vitexin (apigenin-8-*C*-hexoside)/isovitexin	C_21_H_20_O_10_	431.09839	13.51/14.89	flavonoids
hispidulin-*O*-hexoside/isomers	C_22_H_22_O_11_	461.10893	13.63/14.96	flavonoids
kaempferol (or luteolin)-*O*-hexoside/isomers	C_21_H_20_O_11_	447.09331	13.76/14.66	flavonoids
isoquercitrin/quercitrin (quercetin-3-rhamnoside)	C_21_H_20_O_11_	447.09331	13.76/14.66	flavonoids
cynaroside (luteolin-7-hexoside)	C_21_H_20_O_11_	447.09328	13.79	flavonoids
apigenin 7-*O*-glucosylhexoside	C_27_H_30_O_15_	593.15122	13.82	flavonoids
isorhamnetin-3-*O*-hexoside	C_22_H_22_O_12_	477.10387	13.87	flavonoids
isorhamnetin-3-*O*-rutinoside	C_28_H_32_O_16_	623.16178	14	flavonoids
azelaic acid	C_9_H_16_O_4_	187.09761	14.12	dicarboxylic acids
2′,6-dihydroxyflavone	C_15_H_10_O_4_	253.05066	14.67	flavonoids
quercetin	C_15_H_10_O_7_	301.03540	15.22	flavonoids
naringenin	C_15_H_12_O_5_	271.06122	15.65	flavonoids
baptigenin	C_15_H_10_O_6_	285.04046	15.71	flavonoids
kaempferol	C_15_H_10_O_6_	285.04049	16.67	flavonoids
apigenin	C_15_H_10_O_5_	269.04502	16.86	flavonoids
6-methoxyluteolin	C_16_H_12_O_7_	315.05105	16.86	flavonoids
diosmetin	C_16_H_12_O_6_	299.05611	16.93	flavonoids
tricin	C_17_H_14_O_7_	329.06668	17.39/17.69	flavonoids
amentoflavone	C_30_H_18_O_10_	537.08274	18.77	flavonoids
sophoraflavanone G/isomers	C_25_H_28_O_6_	423.18134	19.48	flavonoids
gallic acid	C_7_H_6_O_5_	169.01427	19.66	phenolcarboxylic acids
glycitein	C_16_H_12_O_5_	283.06122	19.66	flavonoids
ginkgetin	C_32_H_22_O_10_	565.11404	21.39	flavonoids

**Table 7:** Qualitative analysis of *Calendulae flos* extract by UHPLC-HRMS/MS.

Compound	Molecular Formula	Adduct Ion (*m*/*z*) Monitored Negative Ion	Retention Time (min)	Phytochemical Class
quinic acid	C_7_H_12_O_6_	191.05611	0.66	cyclohexanecarboxylic acids
daphnin	C_15_H_16_O_9_	339.072155	5.16	coumarins
cichorin	C_15_H_16_O_9_	339.07218	5.18	coumarins
chlorogenic acid	C_16_H_18_O_9_	353.08783	6.25	phenolcarboxylic acids
caffeic acid	C_9_H_8_O_4_	179.03501	6.88	phenolcarboxylic acids
sinapic acid	C_11_H_12_O_5_	223.06122	7.45	phenolcarboxylic acids
lactucopicrin	C_23_H_22_O_7_	409.12930	7.67	sesquiterpenes
caffeoylshikimic acid	C_10_H_15_O_9_	278.06435	7.72	phenolcarboxylic acids
coumaroylquinic acid/isomers	C_16_H_18_O_8_	337.09292	8.18/10.40	phenolcarboxylic acids
syringic acid	C_9_H_10_O_5_	197.04555	9.09	phenolcarboxylic acids
p-coumaric acid	C_9_H_8_O_3_	163.03954	9.13	phenolcarboxylic acids
hyperoside (quercetin-3-hexoside)	C_21_H_20_O_12_	463.08768	9.61	flavonoids
ferulic acid	C_10_H_10_O_4_	193.05066	10.22	phenolcarboxylic acids
robinin	C_33_H_40_O_19_	739.20913	12.42	flavonoids
cynarin (1,5-di-*O*-caffeoylquinic acid)	C_25_H_24_O_12_	515.11950	12.42/13.72	phenolcarboxylic acids
rutin (quercetin-3-rutinoside)	C_27_H_30_O_16_	609.14613	12.56	flavonoids
kaempferol-3-*O*-rutinoside	C_27_H_30_O_15_	593.15122	12.7	flavonoids
quercetin-3-(6-malonyl)-hexoside	C_24_H_22_O_15_	549.08862	13.09	flavonoids
chryosoeriol-7-hexoside	C_22_H_22_O_11_	461.10893	13.38	flavonoids
cynaroside (luteolin-7-hexoside)	C_21_H_20_O_11_	447.09328	13.8	flavonoids
isoquercitrin/quercitrin (quercetin-3-rhamnoside)	C_21_H_20_O_11_	447.09331	13.8	flavonoids
kaempferol (or luteolin)-*O*-hexoside/isomers	C_21_H_20_O_11_	447.09331	13.80/14.56	flavonoids
apigenin-7-*O*-glucosylhexoside	C_27_H_30_O_15_	593.15122	13.85	flavonoids
isorhamnetin-3-*O*-hexoside	C_22_H_22_O_12_	477.10387	13.9	flavonoids
isorhamnetin-3-*O*-rutinoside	C_28_H_32_O_16_	623.16178	14	flavonoids
azelaic acid	C_9_H_16_O_4_	187.09761	14.13	dicarboxylic acids
abscisic acid	C_15_H_20_O_4_	263.12891	14.87	sesquiterpenes
hispidulin-*O*-hexoside/isomers	C_22_H_22_O_11_	461.10893	14.98	flavonoids
quercetin	C_15_H_10_O_7_	301.03540	15.2	flavonoids
naringenin	C_15_H_12_O_5_	271.06122	15.6	flavonoids
procyanidine	C_30_H_26_O_13_	593.13006	16	flavonoids
apigenin	C_15_H_10_O_5_	269.04502	16.88	flavonoids
carnasol	C_20_H_26_O_4_	329.17586	18.89	diterpenes
calenduloside G/isomers	C_42_H_66_O_14_	793.43800	21.02/23.03	triterpenes
calenduloside E/isomers	C_36_H_56_O_9_	631.38518	23.47	triterpenes

**Table 8:** Quantitative analysis by UHPLC-HRMS/MS.

Phytochemical Class	Compound	Quantity (μg/g)		
		SE	GE	CE
phenolcarboxylic acids	caffeic acid	/	3.22	12.86
phenolcarboxylic acids	p-coumaric acid	1241.65	909.27	7.09
phenolcarboxylic acids	syringic acid	722.67	197.84	622.28
phenolcarboxylic acids	chlorogenic acid	/	386.41	20,676.63
phenolcarboxylic acids	ferulic acid	244.36	/	249.23
phenolcarboxylic acids	gallic acid	4472.70	1608.25	189.95
flavonoids	epicatechin	/	918.6	/
flavonoids	genistin	6974.06	/	/
flavonoids	glicitein	375.18	305.1	/
flavonoids	hyperoside	2485.75	999.62	677.76
flavonoids	apigenin	1.87	79.64	/
flavonoids	rutin	104,186.77	3907.47	2165.42
flavonoids	formononetin	6.98	/	/
flavonoids	galangin	291.14	/	/
flavonoids	kaempherol	3263.84	236.96	/
flavonoids	hesperetin	8859.90	/	/
flavonoids	naringenin	14.00	15.12	/
flavonoids	quercetin	46,678.34	4504.66	352.14
flavonoids	isorhamnetin	97,049.32	5032.60	11,286.93
flavonoids	daidzein	18.24	60.51	/
sesquiterpenes	abscinic acid	164.67	151.88	144.76

**Table 9:** Amount of active compounds and antioxidant activity.

Extract	PCA	FLV	PPC	QHPLC	FRAP	DPPH	ABTS
SE	17.14	37.45	28.49	277.05	0.1294	0.0948	0.0715
GE	2.17	2.37	7.23	19.32	0.4806	0.5185	0.1939
CE	3.81	2.01	4.90	36.39	0.4370	0.8068	0.3693

SE = *Sophorae flos* extract; GE = *Ginkgo bilobae folium* extract; CE = *Calendulae flos* extract; PCA = phenolcarboxylic acids content (g chlorogenic acid/100 g extract); FLV = flavonoid content (g rutin/100 g extract); PPC = Polyphenolic content (g tannic acid/100 g extract); QHPLC = polyphenolic content from quantitative UHPLC method (mg/g); FRAP = EC_50_ (mg/mL) from ferric reducing antioxidant power assay (mg/mL); DPPH = IC_50_ from 2,2-diphenyl-1-picryl-hydrazine assay (mg/mL); ABTS = IC_50_ from 2,20-azinobis-3-ethylbenzotiazoline-6-sulfonic acid assay (mg/mL).

**Table 10:** Correlations between quantitative results from UHPLC-HRMS/MS analysis and spectrophotometric analysis.

Correlation	r	R^2^	R^2^ (%)
QHPLC vs. PCA *	0.999	0.998	99.800
QHPLC vs. FLV *	0.998	0.996	99.600
QHPLC vs. PPC	0.989	0.978	97.812

r = Pearson correlation coefficient; R^2^ = coefficient of determination; R^2^ (%) = coefficient of determination as a percentage; * statistically significant for *p* = 0.05.

**Table 11:** Correlations between amount of active compounds and antioxidant activity.

Correlation	r	R^2^	R^2^ (%)
FLV vs. FRAP *	−0.997	0.994	99.400
FLV vs. DPPH	−0.982	0.964	96.432
FLV vs. ABTS	−0.924	0.853	85.377
PCA vs. FRAP *	−0.999	0.998	99.800
PCA vs. DPPH	−0.956	0.913	91.393
PCA vs. ABTS	−0.878	0.770	77.088
PPC vs. FRAP *	−0.988	0.976	97.614
PPC vs. DPPH	−0.994	0.988	98.803
PPC vs. ABTS	−0.952	0.906	90.630
QHPLC vs. FRAP *	−1.000	1.000	100.000
QHPLC vs. DPPH	−0.967	0.935	93.509
QHPLC vs. ABTS	−0.896	0.802	80.281

r = Pearson correlation coefficient; R^2^ = coefficient of determination; R^2^ (%) = coefficient of determination as a percentage; * statistically significant for *p* = 0.05.

## Data Availability

Not applicable.

## Supplementary Materials

The following supporting information can be downloaded at: https://www.mdpi.com/article/10.3390/molecules28093668/s1, Figure S1: Rutin–*Sophorae flos* extract spectrogram ESI+, water sample; Figure S2: Quercetin–*Sophorae flos* extract spectrogram ESI+, water sample; Figure S3: Isorhamnetin–*Sophorae flos* extract spectrogram ESI+, water sample; Figure S4: Kaempferol/fisetin– *Sophorae flos* extract spectrogram ESI+, water sample; Figure S5: Isoquercetin–*Sophorae flos* extract spectrogram ESI+, water sample; Figure S6: Sophoricoside–*Sophorae flos* extract spectrogram ESI+, water sample; Figure S7: Kaikasaponin I–*Sophorae flos* extract spectrogram ESI+, methanol sample; Figure S8: Kaikasaponin III– *Sophorae flos* extract spectrogram ESI+, methanol sample; Figure S9: Rutin–*Ginkgo bilobae folium* extract spectrogram ESI+, water sample; Figure S10: Bilobalide–*Ginkgo bilobae folium* extract spectrogram ESI+, water sample; Figure S11: Quercetin–*Ginkgo bilobae folium* extract spectrogram ESI+, water sample; Figure S12: Kaempferol–*Ginkgo bilobae folium* extract spectrogram ESI+, water sample; Figure S13: Ginkgolide A-*Ginkgo bilobae folium* extract spectrogram ESI+, water sample; Figure S14: Ginkgolide B-*Ginkgo bilobae folium* extract spectrogram ESI+, water sample; Figure S15: Ginkgolide C-*Ginkgo bilobae folium* extract spectrogram ESI+, water sample; Figure S16: Isoquercitrin–*Ginkgo bilobae folium* extract spectrogram ESI+, methanol sample; Figure S17: Isorhamnetin–*Ginkgo bilobae folium* extract spectrogram ESI+, methanol sample; Figure S18: Catechin–*Ginkgo bilobae folium* extract spectrogram ESI+, methanol sample; Figure S19: Bilobetin–*Ginkgo bilobae folium* extract spectrogram, ESI+, methanol sample; Figure S20: Caffeic acid–*Ginkgo bilobae folium* extract spectrogram, ESI+, methanol sample; Figure S21: Ginkgolide B–*Ginkgo bilobae folium* extract spectrogram, ESI–, methanol sample; Figure S22: Bilobalide–*Ginkgo bilobae folium* extract spectrogram, ESI–, methanol sample; Figure S23: Rutin–*Calendulae flos* extract spectrogram, ESI+, water sample; Figure S24: Calendoflavoside/narcissin–*Calendulae flos* extract spectrogram, ESI+, water sample; Figure S25: Thyphaneoside–*Calendulae flos* extract spectrogram, ESI+, water sample; Figure S26: Isorhamnetin–*Calendulae flos* extract spectrogram, ESI+, water sample; Figure S27: Isoquercitrin–*Calendulae flos* extract spectrogram, ESI+, water sample; Figure S28: Quercetin–*Calendulae flos* extract spectrogram, ESI+, water sample; Figure S29: Quantitative spectrophotometric analysis–boxplot for *Sophorae flos* extract; Figure S30: Quantitative spectrophotometric analysis–boxplot for *Ginkgo bilobae folium* extract; Figure S31: Quantitative spectrophotometric analysis–boxplot for *Calendulae flos* extract; Figure S32: Regression trendlines and equations–FRAP assay; Figure S33: Regression trendlines and equations–DPPH assay; Figure S34: Regression trendlines and equations–ABTS assay; Figure S35: Superposition of predicted conformations for MMPs inhibitors on experimentally determined conformations; Figure S36: Chlorogenic acid standard curve; Figure S37: Rutin standard curve; Figure S38: Tannic acid standard curve; Table S1: UHPLC-HRMS/MS general screening; Table S2: Correlation between flavonoid content (FLV) and antioxidant methods; Table S3: Correlation between phenolcarboxylic acids content (PCA) and antioxidant methods; Table S4: Correlation between polyphenolic content (PPC) and antioxidant methods; Table S5: Correlation between UHPLC-HRMS/MS method (QHPLC), antioxidant methods and spectrophotometric methods; Table S6: Molecular docking results after screening the selected phytochemicals.
