# Supplementary material for: Outlook on Chronic Venous Disease Treatment: Phytochemical Screening, In Vitro Antioxidant Activity and In Silico Studies for Three Vegetal Extracts
Source: Molecules. 2023 Apr 23;28(9):3668. doi: 10.3390/molecules28093668 (PMC10180301; doi:10.3390/molecules28093668)
Supplement: Supplementary file 1 [file molecules-28-03668-s001.zip › molecules-2350047-supplementary.pdf]

Article

Outlook on Chronic Venous Disease Treatment: Phytochemical Screening, In Vitro Antioxidant Activity and In Silico Studies for Three Vegetal Extracts

Supplementary Materials

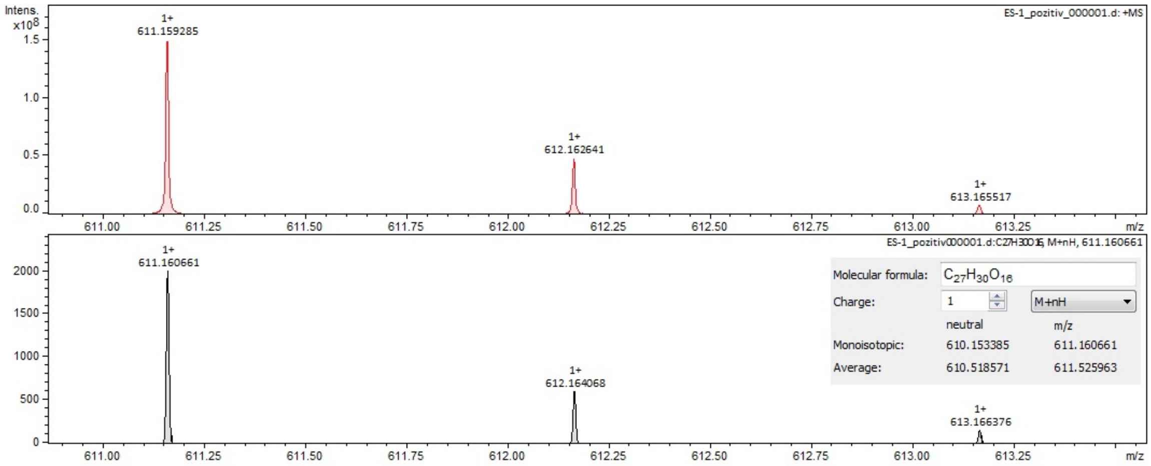

Figure S1. Rutin – *Sophorae flos* extract spectrogram ESI+, water sample.

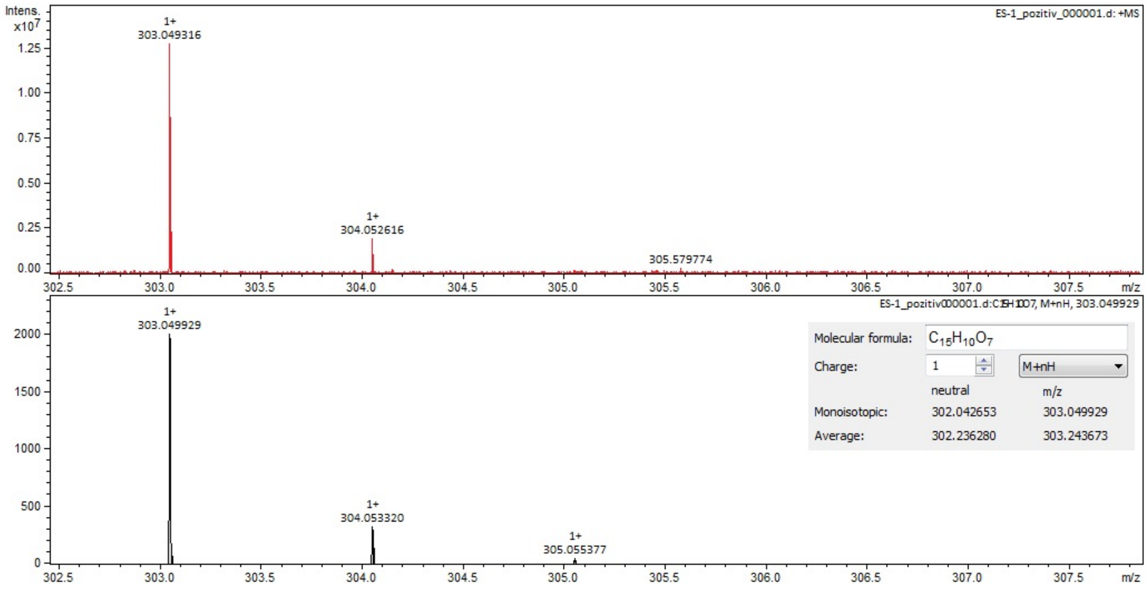

Figure S2. Quercetin – *Sophorae flos* extract spectrogram ESI+, water sample.

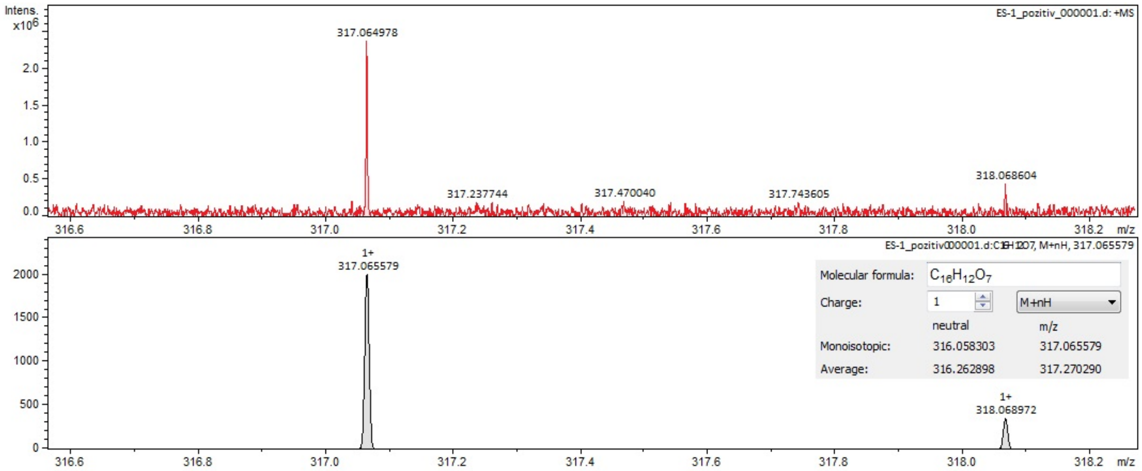

Figure S3. Isorhamnetin – *Sophorae flos* extract spectrogram ESI+, water sample.

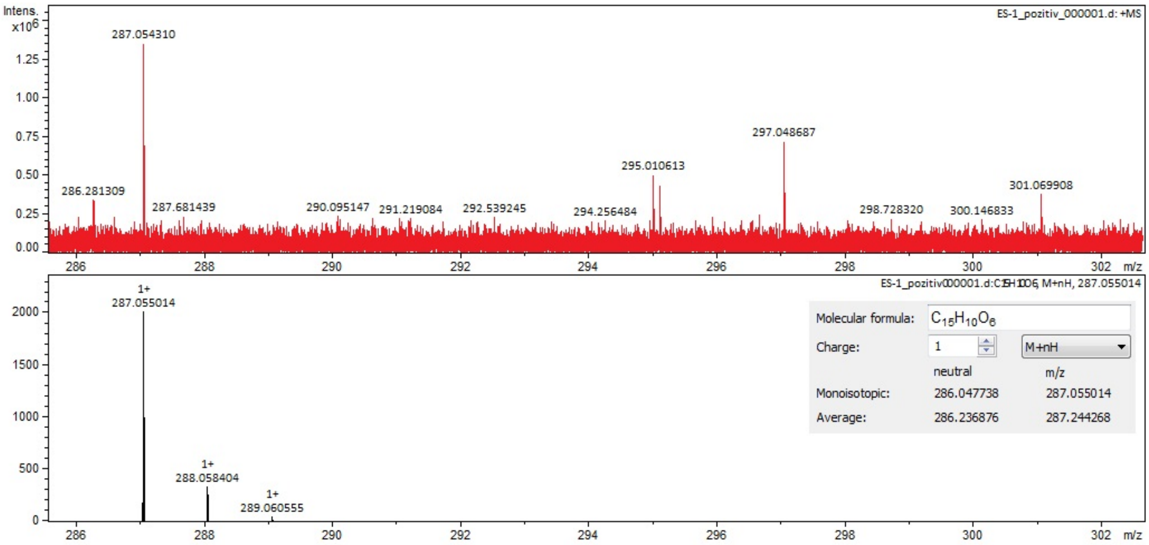

Figure S4. Kaempferol/fisetin– *Sophorae flos* extract spectrogram ESI+, water sample.

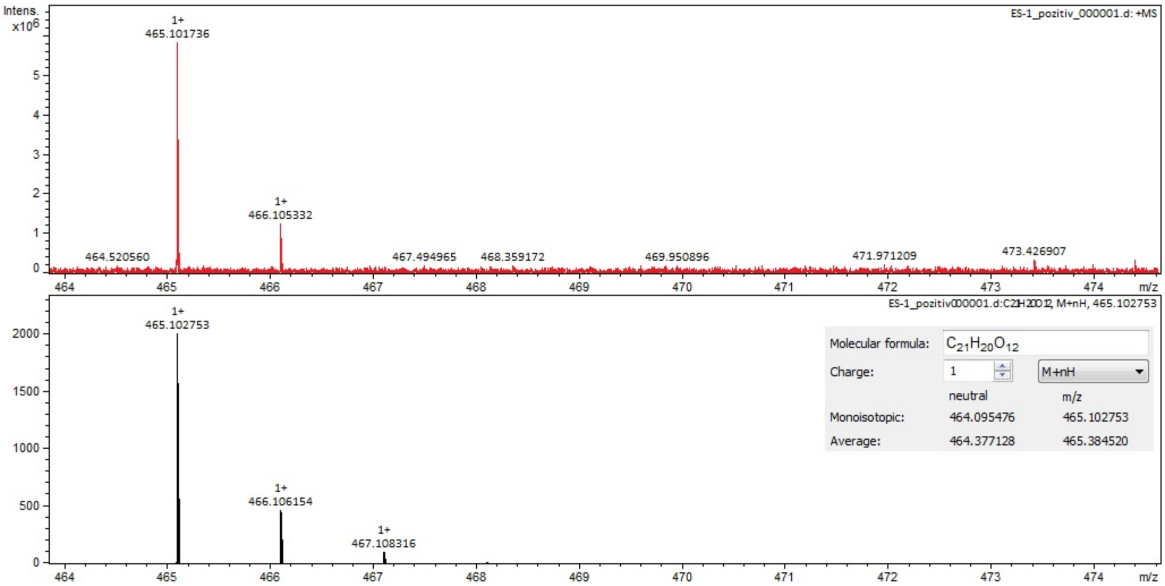

Figure S5. Isoquercetin – *Sophorae flos* extract spectrogram ESI+, water sample.

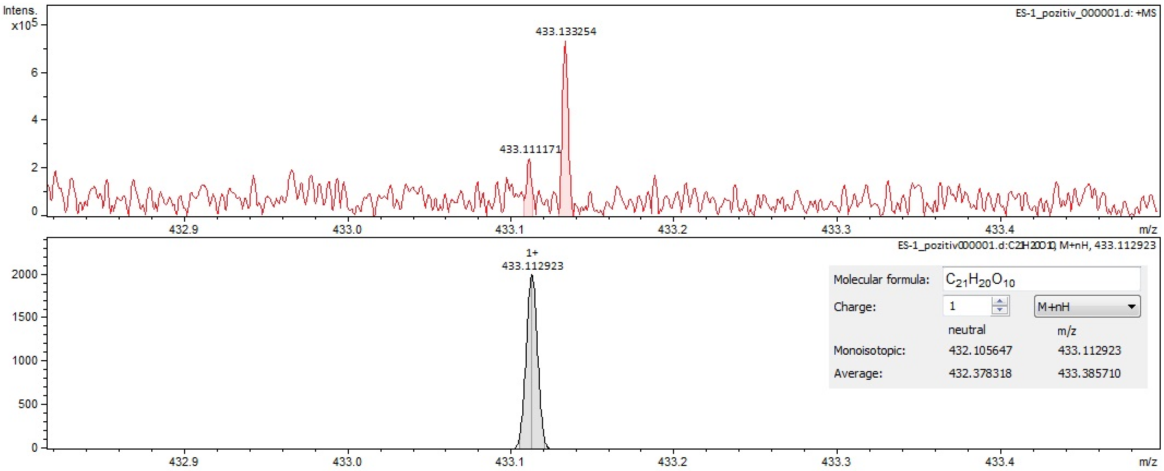

Figure S6. Sophoricoside – *Sophorae flos* extract spectrogram ESI+, water sample.

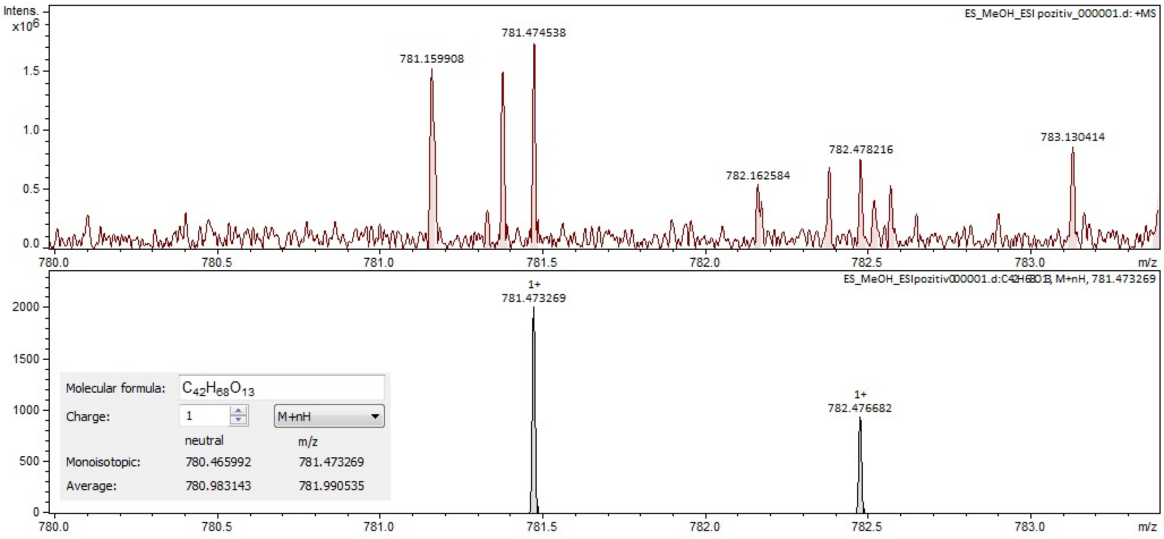

Figure S7. Kaikasaponin I – *Sophorae flos* extract spectrogram ESI+, methanol sample.

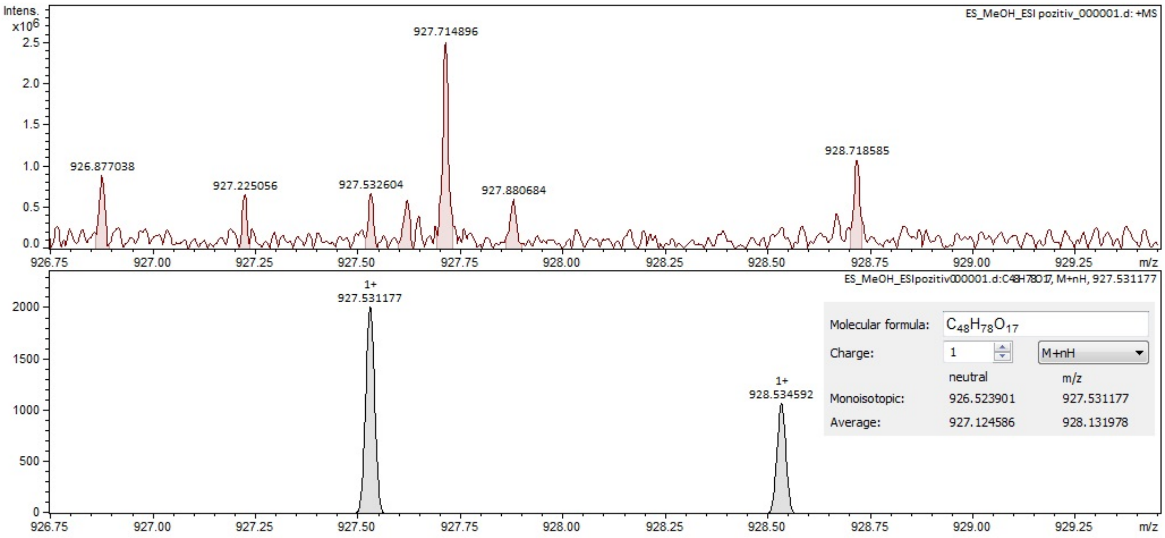

Figure S8. Kaikasaponin III– *Sophorae flos* extract spectrogram ESI+, methanol sample.

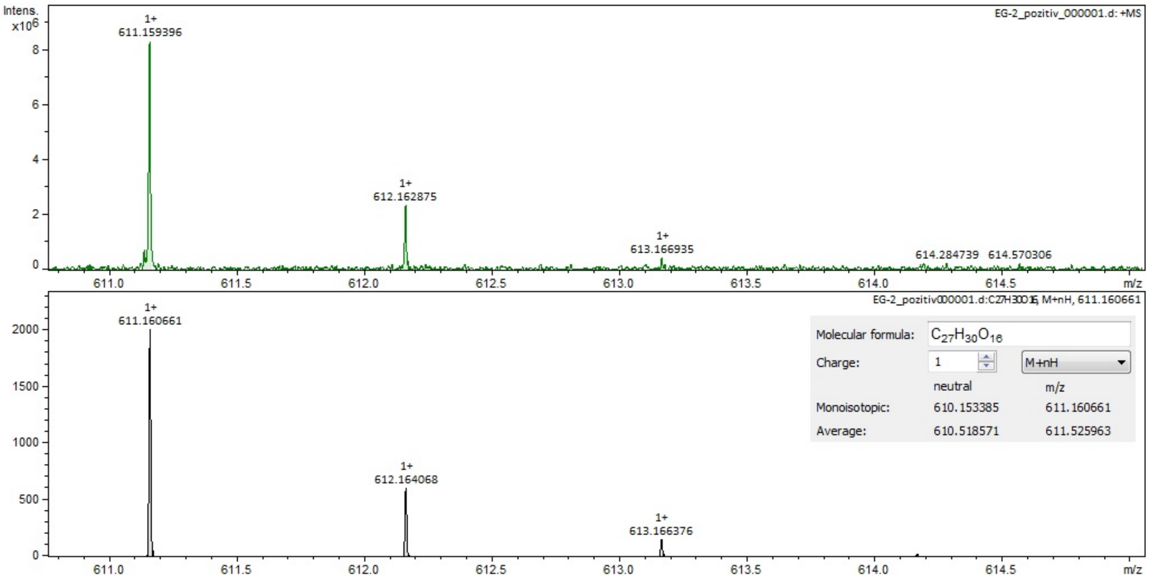

Figure S9. Rutin – *Ginkgo bilobae folium* extract spectrogram ESI+, water sample.

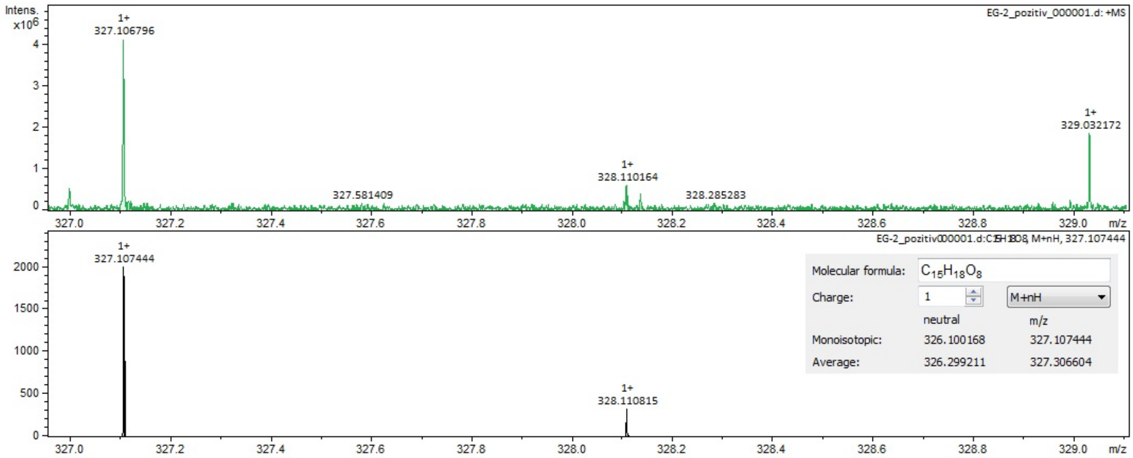

Figure S10. Bilobalide – *Ginkgo bilobae folium* extract spectrogram ESI+, water sample.

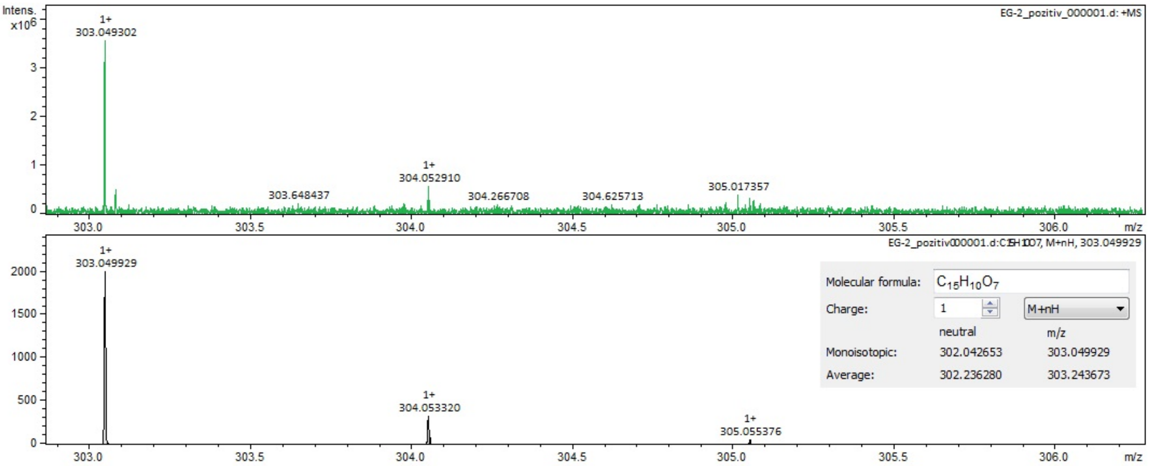

Figure S11. Quercetin – *Ginkgo bilobae folium* extract spectrogram ESI+, water sample.

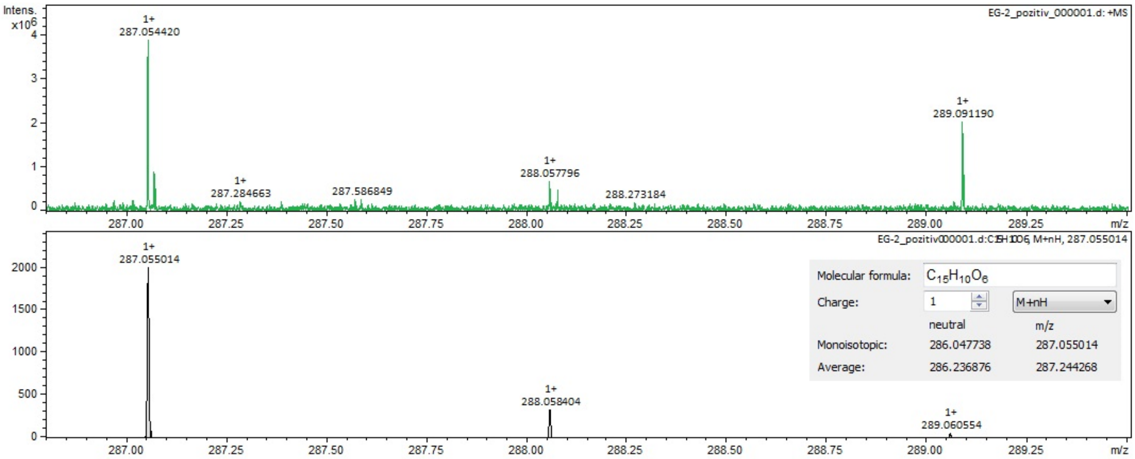

Figure S12. Kaempferol – *Ginkgo bilobae folium* extract spectrogram ESI+, water sample.

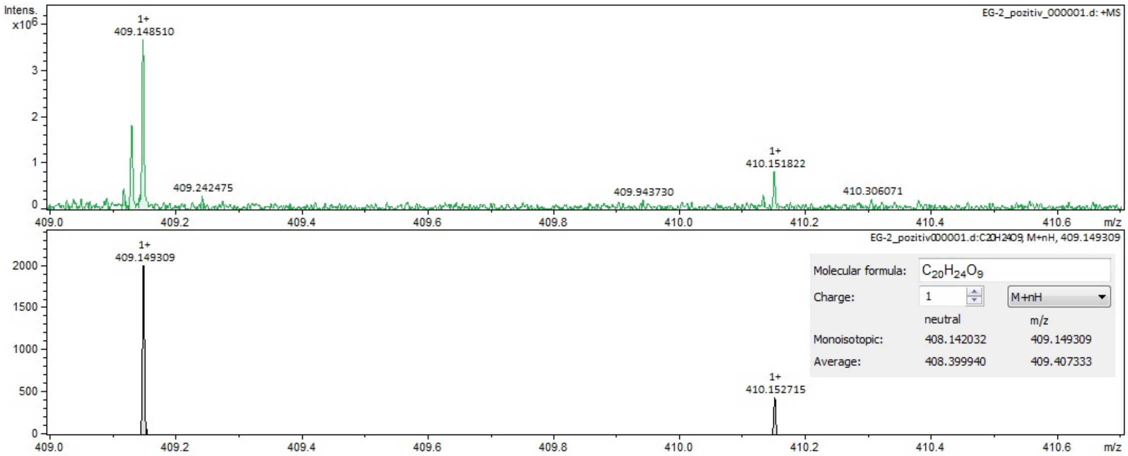

Figure S13. Ginkgolide A - *Ginkgo bilobae folium* extract spectrogram ESI+, water sample.

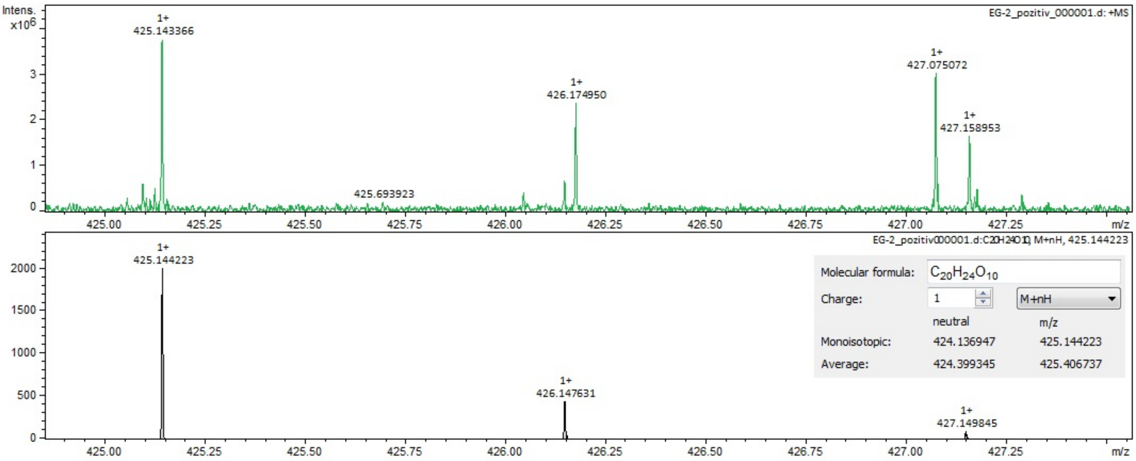

Figure S14. Ginkgolide B - *Ginkgo bilobae folium* extract spectrogram ESI+, water sample.

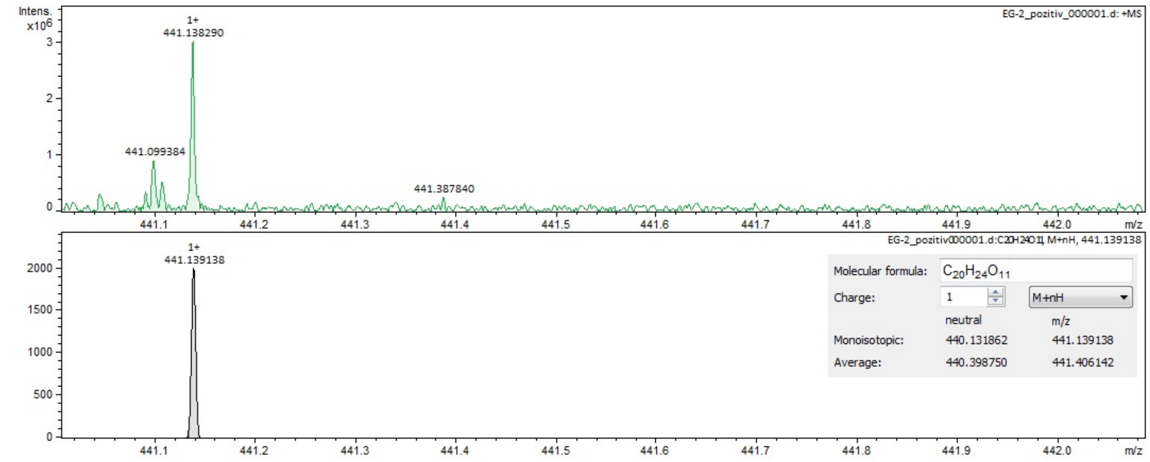

Figure S15. Ginkgolide C– *Ginkgo bilobae folium* extract spectrogram ESI+, water sample.

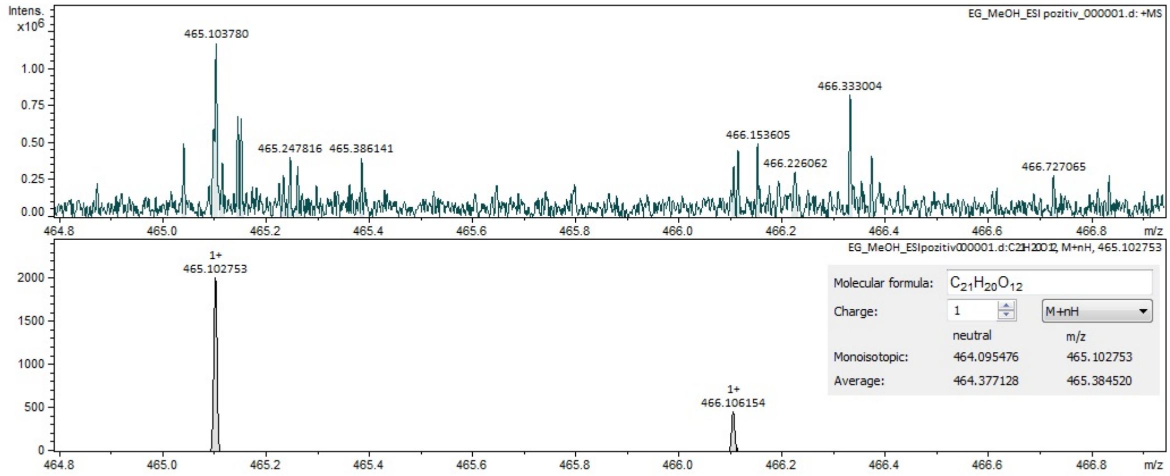

Figure S16. Isoquercitrin – *Ginkgo bilobae folium* extract spectrogram ESI+, methanol sample.

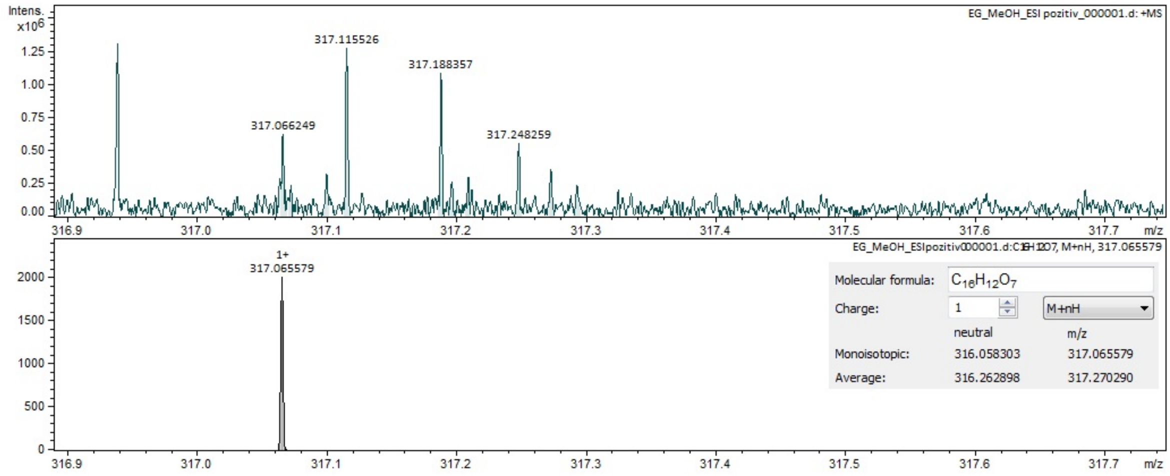

Figure S17. Isorhamnetin – *Ginkgo bilobae folium* extract spectrogram ESI+, methanol sample.

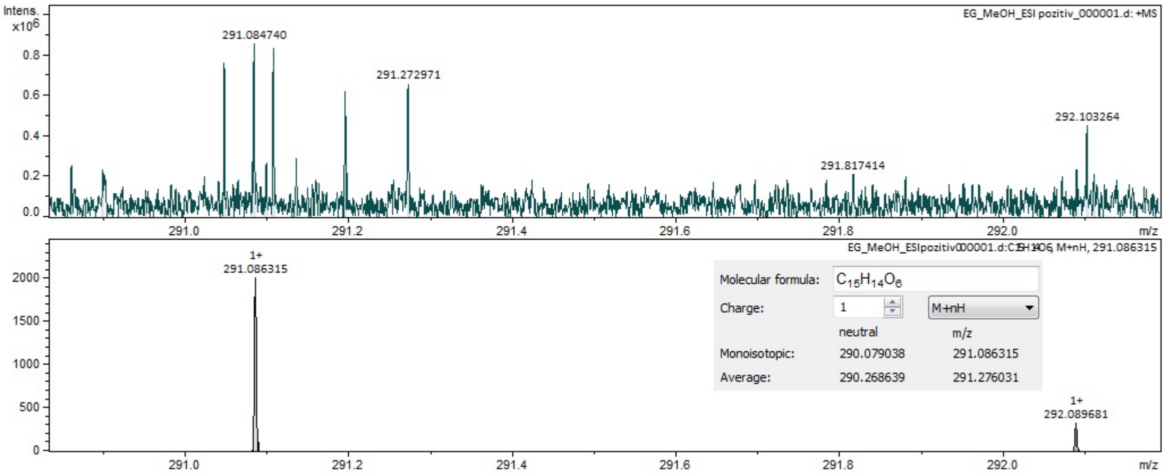

Figure S18. Catechin – *Ginkgo bilobae folium* extract spectrogram ESI+, methanol sample.

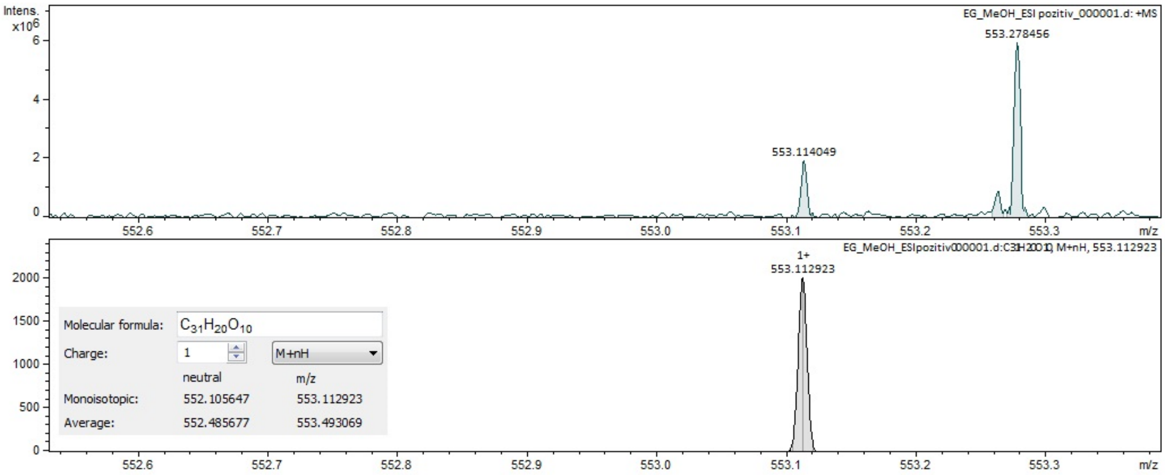

Figure S19. Bilobetin – *Ginkgo bilobae folium* extract spectrogram, ESI+, methanol sample.

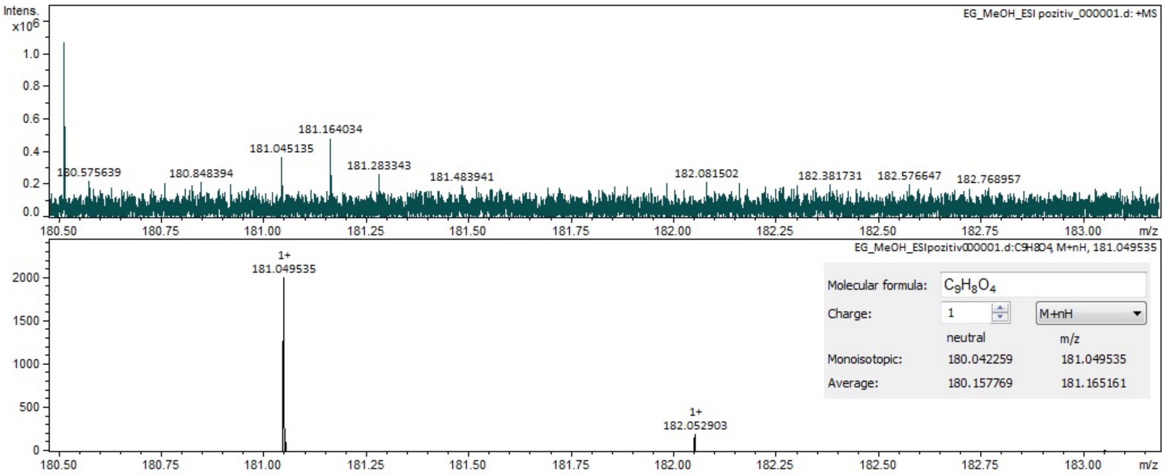

Figure S20. Caffeic acid – *Ginkgo bilobae folium* extract spectrogram, ESI+, methanol sample.

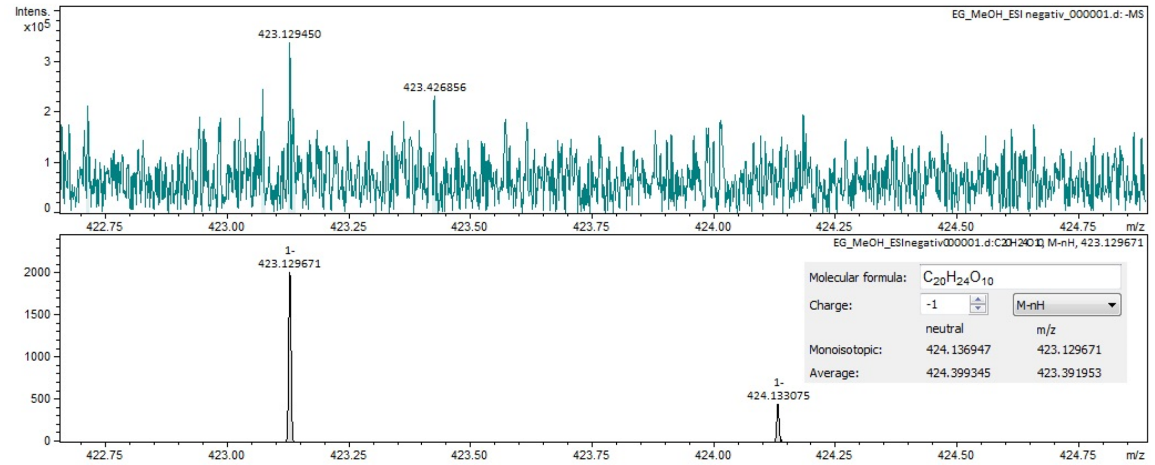

Figure S21. Ginkgolide B – *Ginkgo bilobae folium* extract spectrogram, ESI–, methanol sample.

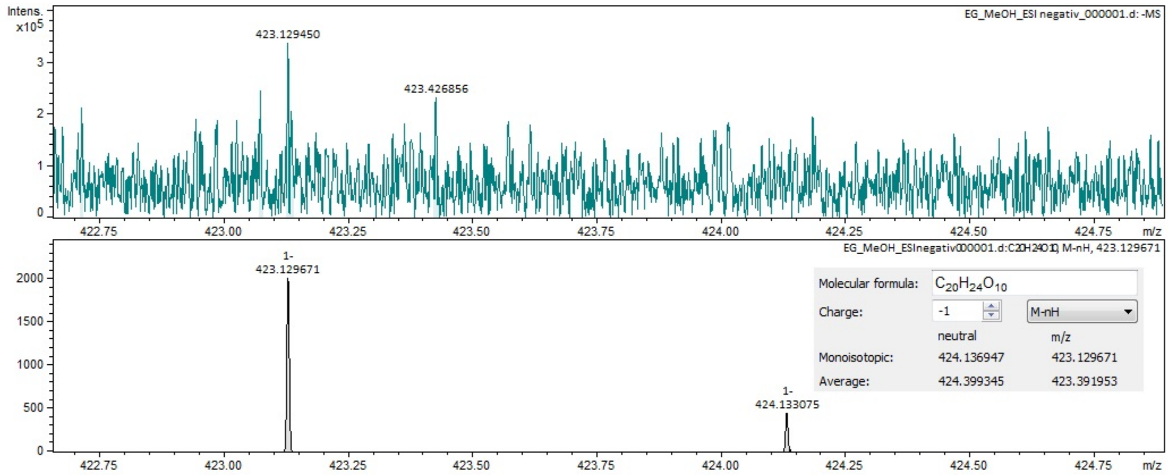

Figure S22. Bilobalide – *Ginkgo bilobae folium* extract spectrogram, ESI–, methanol sample.

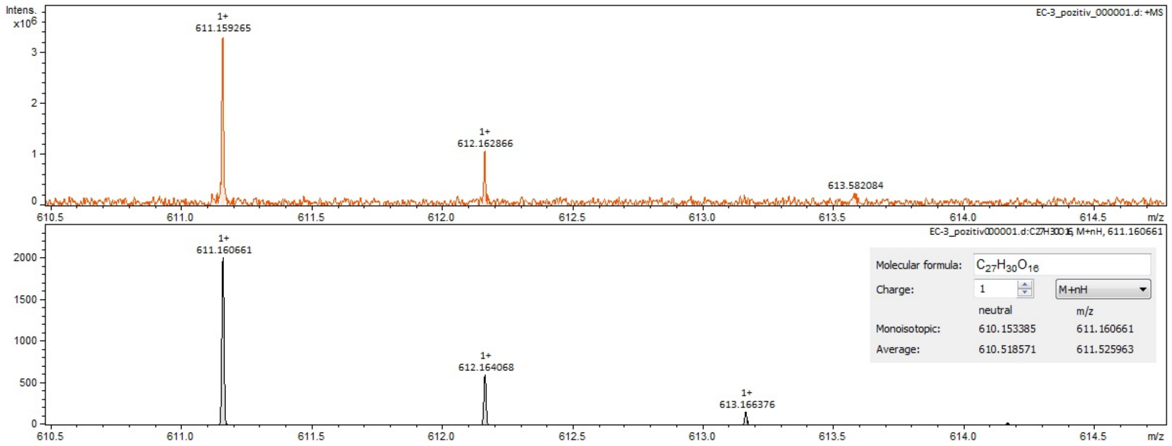

Figure S23. Rutin – *Calendulae flos* extract spectrogram, ESI+, water sample.

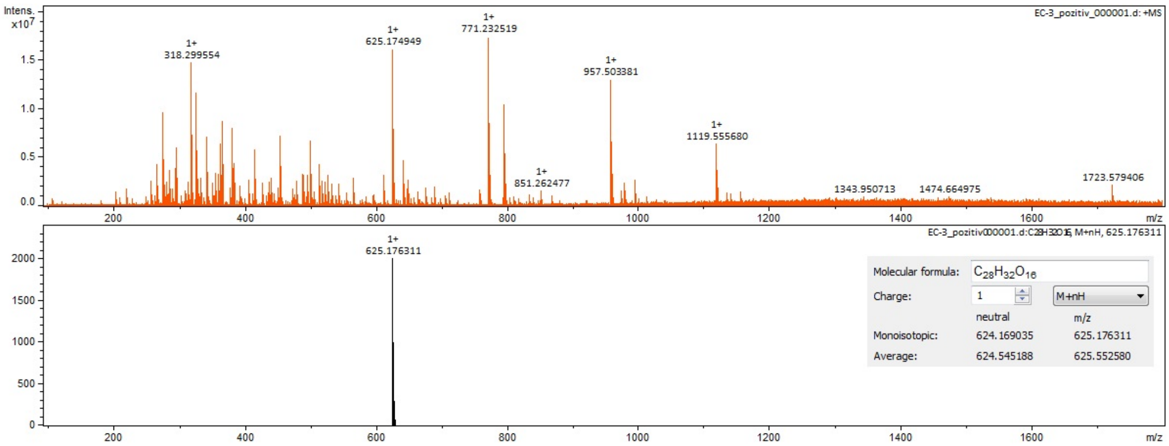

Figure S24. Calendoflavoside/narcissin – *Calendulae flos* extract spectrogram, ESI+, water sample.

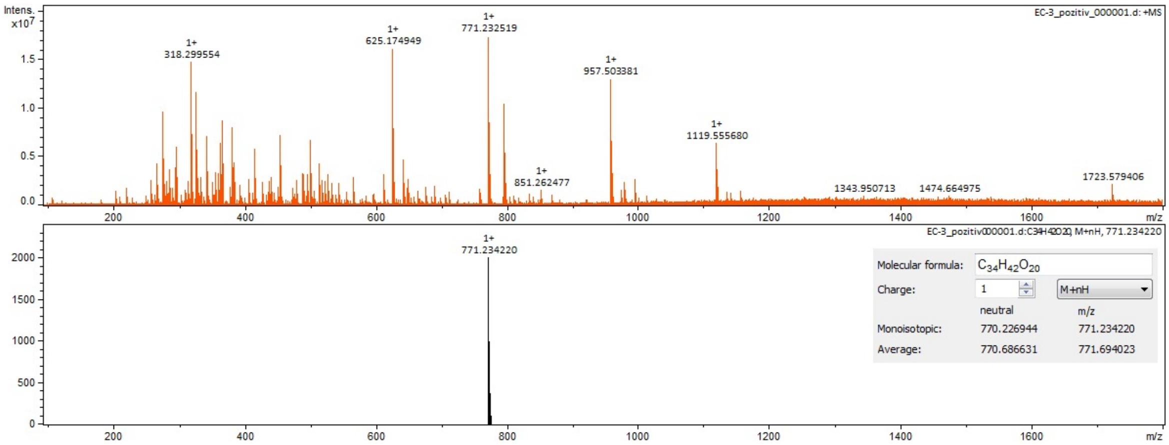

Figure S25. Thyphaneoside – *Calendulae flos* extract spectrogram, ESI+, water sample.

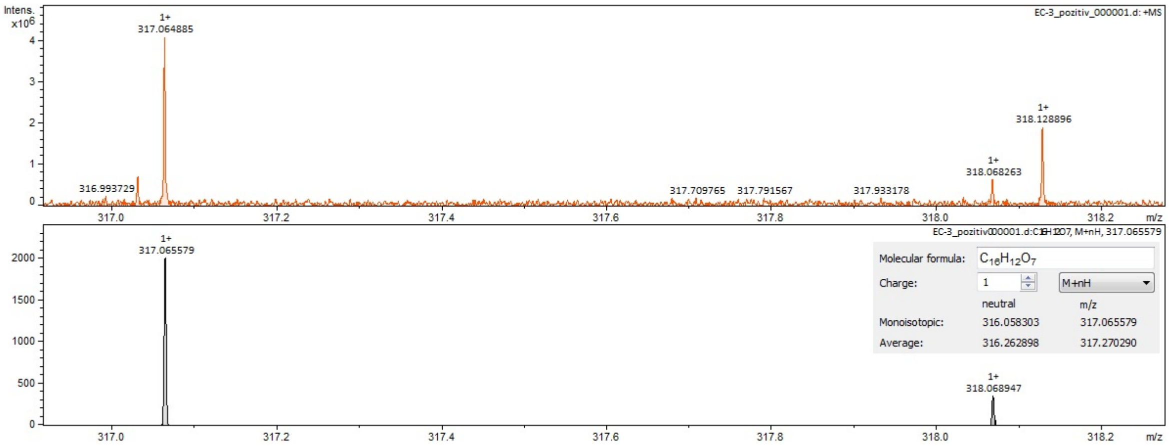

Figure S26. Isorhamnetin – *Calendulae flos* extract spectrogram, ESI+, water sample.

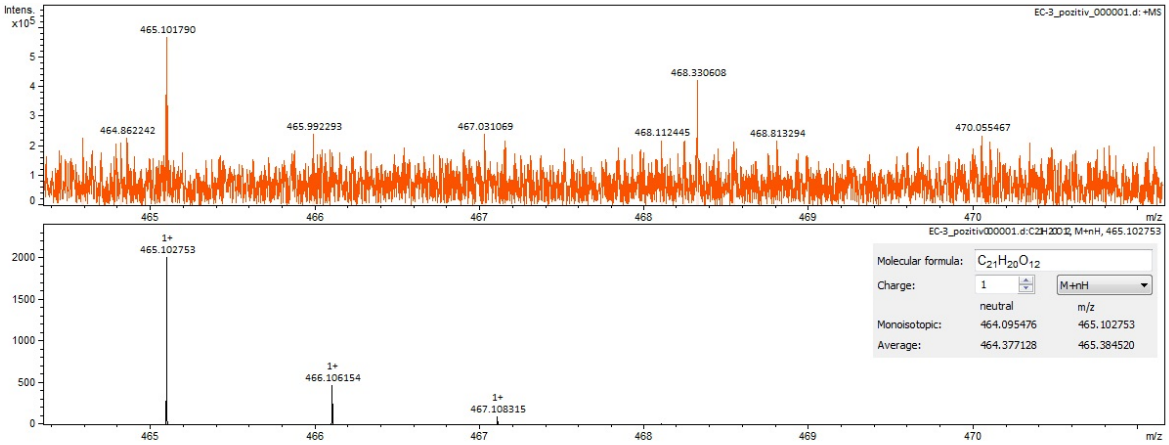

Figure S27. Isoquercitrin – *Calendulae flos* extract spectrogram, ESI+, water sample.

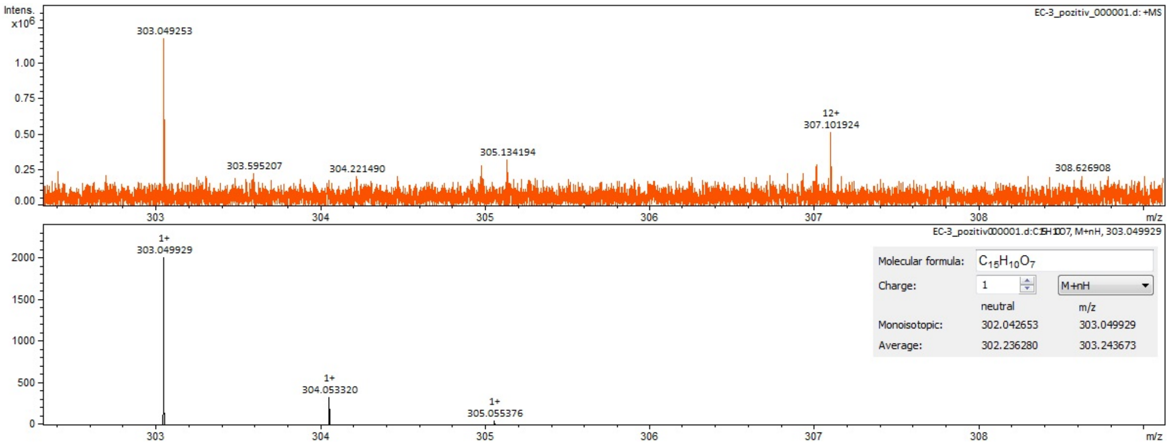

Figure S28. Quercetin – *Calendulae flos* extract spectrogram, ESI+, water sample.

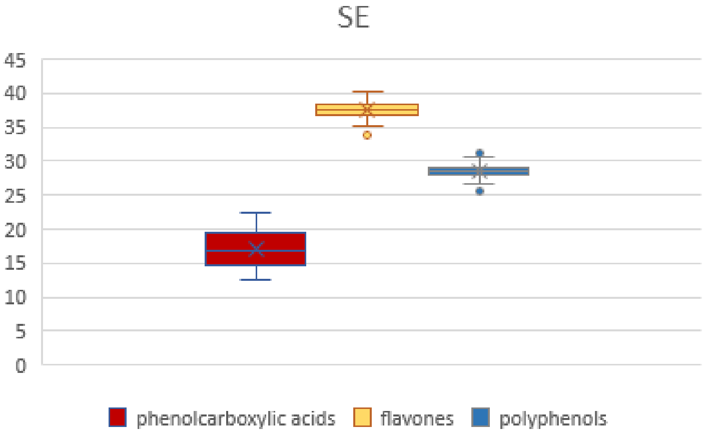

Figure S29. Quantitative spectrophotometric analysis – boxplot for *Sophorae flos* extract.

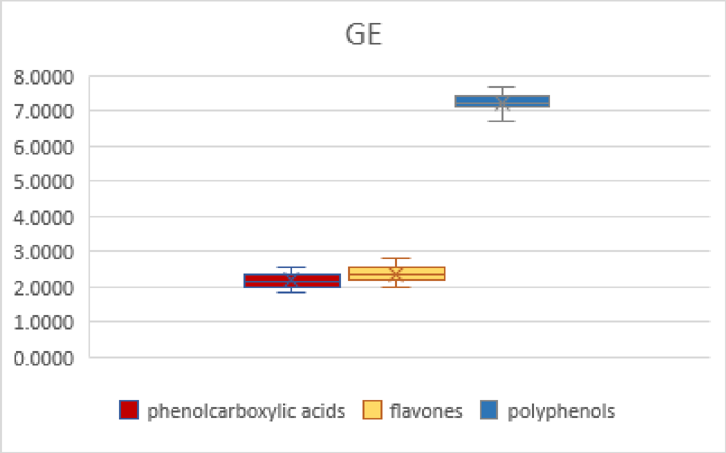

Figure S30. Quantitative spectrophotometric analysis – boxplot for *Ginkgo bilobae folium* extract.

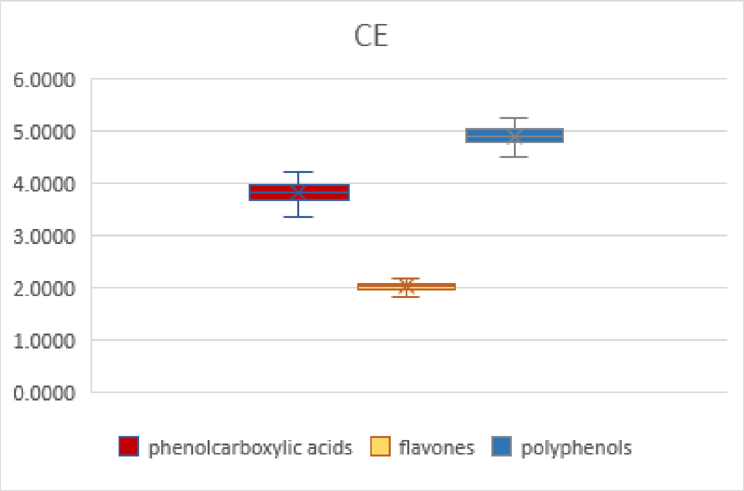

Figure S31. Quantitative spectrophotometric analysis – boxplot for *Calendulae flos* extract.

Table S1. UHPLC-HRMS/MS general screening.

Nr.	Phytochemical class	Compound	Molecular formula	Adduct Ion (*m/z*) Monitored Negative Ion	Retention time (min)		
					**SE**	**GE**	**CE**
1	carboxylic acids	folinic acid	$C_{20}H_{23}N_7O_7$	472.15864	/	/	/
2	cyclohexancarboxylic acids	quinic acid	$C_7H_{12}O_6$	191.05611	0.66	0.66	0.66
3	Cyclohexencarboxylic acids	perillic acid	$C_{10}H_{14}O_2$	165.09210	/	/	/
4	dicarboxylic acids	azelaic acid	$C_9H_{16}O_4$	187.09761	14.13	14.12	14.1
5	dicarboxylic acids	malic acid	$C_4H_6O_5$	133.01427	/	/	/
6	phenolcarboxylic acids	dihydro-o-coumaric acid	$C_9H_{10}O_3$	165.05574	/	/	/
7	phenolcarboxylic acids	gallic acid	$C_7H_6O_5$	169.01427	/	19.66	/
8	phenolcarboxylic acids	chlorogenic acid	$C_{16}H_{18}O_9$	353.08783	6.25	6.23	/
9	phenolcarboxylic acids	caffeic acid	$C_9H_8O_4$	179.03501	6.88	/	/

Nr.	Phytochemical class	Compound	Molecular formula	Adduct Ion (*m/z*) Monitored Negative Ion	Retention time (min)		
					SE	GE	CE
10	phenolcarboxylic acids	ferulic acid	$C_{10}H_{10}O_4$	193.05066	10.22	/	/
11	phenolcarboxylic acids	ellagic acid	$C_{14}H_6O_8$	300.99899	/	/	/
12	phenolcarboxylic acids	p-coumaric acid	$C_9H_8O_3$	163.03954	9.13	9.09	9.09
13	phenolcarboxylic acids	syringic acid	$C_9H_{10}O_5$	197.04555	9.09	8.86	8.86
14	phenolcarboxylic acids	cynarin (1,5-di-*O*-caffeoylquinic acid)	$C_{25}H_{24}O_{12}$	515.11950	12.42/13.72	12.37	/
15	phenolcarboxylic acids	rosmarinic acid	$C_{18}H_{16}O_8$	359.07727	/	/	/
16	phenolcarboxylic acids	carnosic acid	$C_{20}H_{28}O_4$	331.19151	/	/	/
17	phenolcarboxylic acids	neochlorogenic acid	$C_{16}H_{18}O_9$	353.08783	/	/	/
18	phenolcarboxylic acids	salvianolic acid B	$C_{36}H_{30}O_{16}$	717.14611	/	/	/
19	phenolcarboxylic acids	caftaric acid	$C_{13}H_{12}O_9$	311.04085	/	/	/
20	phenolcarboxylic acids	cichoric acid	$C_{22}H_{18}O_{12}$	473.07257	/	/	/
21	phenolcarboxylic acids	coumanoylquinic acid/ isomers	$C_{16}H_{18}O_8$	337.09292	8.18/1.04	/	/
22	phenolcarboxylic acids	anisic acid (4-methoxybenzoic acid)	$C_8H_8O_3$	151.04009	/	/	/
23	phenolcarboxylic acids	sinapic acid	$C_{11}H_{12}O_5$	223.06122	7.45	/	/
24	phenolcarboxylic acids	hydroxyferulic acid	$C_{16}H_{20}O_{10}$	371.09839	/	6.79	6.77
25	phenolcarboxylic acids	caffeoylshikimic acid	$C_{10}H_{15}O_9$	278.06435	7.72	7.7	7.65
26	carotenoids	violaxantin	$C_{40}H_{56}O_4$	599.41061	/	/	/
27	carotenoids	taraxantin	$C_{40}H_{56}O_3$	583.41569	/	/	/
28	coumarins	cichorin	$C_{15}H_{16}O_9$	339.07218	5.18	/	/
29	coumarins	aesculetin/isomers	$C_9H_6O_4$	177.01936	/	/	/
30	coumarins	scopoletin	$C_{10}H_8O_4$	191.03501	/	/	/
31	coumarins	esculetin-7-hexoside	$C_{15}H_{16}O_9$	339.07218	/	/	/
32	coumarins	coumarin	$C_9H_6O_2$	145.02953	/	/	/
33	coumarins	dihydrocoumarin	$C_9H_8O_2$	147.04518	/	/	/
34	coumarins	7-hydroxycoumarin	$C_9H_6O_3$	161.02444	/	/	/
35	diterpenes	carnasol	$C_{20}H_{26}O_4$	329.17586	18.89	/	/
36	diterpenes	rosmanol/epirosmanol	$C_{20}H_{26}O_5$	345.17077	/	/	/
37	diterpenes	rosmadial/isomers	$C_{20}H_{24}O_5$	343.15510	/	/	/
38	diterpenes	rosmaridiphenol	$C_{20}H_{28}O_3$	315.19657	/	/	/
39	diterpenes	rosmanol methyl ether	$C_{21}H_{28}O_5$	359.18640	/	/	/
40	diterpenes	carnasic acid quinone	$C_{20}H_{25}O_4$	328.16803	/	/	/
41	esters	coniferyl ferulate	$C_{20}H_{20}O_6$	355.11874	/	/	/
42	phenols	coniferyl	$C_{10}H_{12}O_3$	179.07139	/	/	/
43	flavonoids	catechin	$C_{15}H_{14}O_6$	289.07176	/	/	/
44	flavonoids	epicatechin	$C_{15}H_{14}O_6$	289.07176	/	8.01	/
45	flavonoids	quercetin	$C_{15}H_{10}O_7$	301.03540	15.2	15.22	15.15
46	flavonoids	rutin (quercetin-3-rutinoside)	$C_{27}H_{30}O_{16}$	609.14613	12.56	12.6	12.58
47	flavonoids	apigenin	$C_{15}H_{10}O_5$	269.04502	16.88	16.86	16.83
48	flavonoids	kaempferol	$C_{15}H_{10}O_6$	285.04049	/	16.67	16.62
49	flavonoids	6-methoxyluteolin	$C_{16}H_{12}O_7$	315.05105	/	16.86	16.85
50	flavonoids	naringenin	$C_{15}H_{12}O_5$	271.06122	15.6	15.65	15.63

Nr.	Phytochemical class	Compound	Molecular formula	Adduct Ion (m/z) Monitored Negative Ion	Retention time (min)		
					SE	GE	CE
51	flavonoids	naringin	$C_{27}H_{32}O_{14}$	579.17185	/	12.93	13
52	flavonoids	hesperitin	$C_{16}H_{14}O_{6}$	301.07179	/	/	15.71
53	flavonoids	pinostrobin	$C_{16}H_{14}O_{4}$	269.08196	/	/	/
54	flavonoids	pinocembrin	$C_{15}H_{12}O_{4}$	255.06631	/	/	/
55	flavonoids	chrysin	$C_{15}H_{10}O_{4}$	253.05066	/	/	/
56	flavonoids	myricetin	$C_{15}H_{10}O_{8}$	317.03032	/	/	7.82
57	flavonoids	galangin	$C_{15}H_{10}O_{5}$	269.04557	/	/	/
58	flavonoids	hyperoside (quercetin-3-hexoside)	$C_{21}H_{20}O_{12}$	463.08768	9.61	9.64	9.63
59	flavonoids	genistin	$C_{21}H_{20}O_{10}$	431.09837	/	/	12.96
60	flavonoids	genistein	$C_{15}H_{10}O_{5}$	269.04502	/	/	/
61	flavonoids	daidzin	$C_{21}H_{20}O_{9}$	415.10348	/	/	/
62	flavonoids	daidzein	$C_{15}H_{10}O_{4}$	253.05066	/	/	/
63	flavonoids	2',6-dihydroxyflavone	$C_{15}H_{10}O_{4}$	253.05066	/	14.67	/
64	flavonoids	ononin	$C_{22}H_{22}O_{9}$	429.11913	/	/	/
65	flavonoids	formononetin	$C_{16}H_{12}O_{4}$	267.06631	/	/	17.51
66	flavonoids	glycitein	$C_{16}H_{12}O_{5}$	283.06122	/	19.66	19.65
67	flavonoids	coumestrol	$C_{15}H_{8}O_{5}$	267.02990	/	/	/
68	flavonoids	coumestrol-3-*O*-hexoside	$C_{21}H_{18}O_{10}$	429.08274	/	/	/
69	flavonoids	biochanin A	$C_{16}H_{12}O_{5}$	283.06122	/	/	/
70	flavonoids	sissotrin (biochanin A7-*O*-β-D-hexoside)	$C_{22}H_{22}O_{10}$	445.11404	/	/	12.50/15.91
71	flavonoids	5,7-dihydroxy-2'-methoxyflavone	$C_{22}H_{12}O_{5}$	283.06122	/	/	/
72	flavonoids	calycosin (3'-hydroxy-formononetin)	$C_{16}H_{12}O_{5}$	283.06122	/	/	/
73	flavonoids	irilone	$C_{16}H_{10}O_{6}$	297.04049	/	/	/
74	flavonoids	baptigenin	$C_{15}H_{10}O_{6}$	285.04046	/	15.71	/
75	flavonoids	pseudobaptigenin	$C_{16}H_{10}O_{5}$	281.04557	/	/	17.36
76	flavonoids	pratensein	$C_{16}H_{12}O_{6}$	299.05614	/	/	/
77	flavonoids	afrormosin	$C_{17}H_{14}O_{5}$	297.07687	/	/	16.39
78	flavonoids	tectorigenin	$C_{16}H_{12}O_{6}$	299.05611	/	/	/
79	flavonoids	alfalone	$C_{17}H_{14}O_{5}$	297.07687	/	/	/
80	flavonoids	irisolidone	$C_{17}H_{14}O_{6}$	313.07179	/	/	/
81	flavonoids	medicarpin	$C_{16}H_{14}O_{4}$	269.08196	/	/	/
82	flavonoids	liquiritigenin/isoliquiritigenin	$C_{15}H_{12}O_{4}$	255.06631	/	/	/
83	flavonoids	kaempferol-3-*O*- rutinoside	$C_{27}H_{30}O_{15}$	593.15122	12.7	/	/
84	flavonoids	kaempferol (or luteolin)-*O*-hexoside/ isomers	$C_{21}H_{20}O_{11}$	447.09331	13.8/14.56	13.76/14.66	13.75
85	flavonoids	isorhamnetin-3-*O*-hexoside	$C_{22}H_{22}O_{12}$	477.10381	/	5.69	/
86	flavonoids	vitexin (apigenin-8- *C*-hexoside)/isovitexin	$C_{21}H_{20}O_{10}$	431.09839	/	13.51/14.89	/
87	flavonoids	apigetrin (apigenin -7-hexoside)	$C_{21}H_{20}O_{10}$	431.09839	/	/	/
88	flavonoids	chryosoeriol	$C_{16}H_{12}O_{6}$	299.05614	/	/	/
89	flavonoids	chryosoeriol-7-hexoside	$C_{22}H_{22}O_{11}$	461.10893	13.38	13.33/14.96	13.4
90	flavonoids	tricin	$C_{17}H_{14}O_{7}$	329.06668	/	17.39/17.69	/
91	flavonoids	cynaroside (luteolin-7-hexoside)	$C_{21}H_{20}O_{11}$	447.09328	13.8	13.79	13.75
92	flavonoids	apigenin-7-*O*-glucosylhexoside	$C_{27}H_{30}O_{15}$	593.15122	13.85	13.82	13.8
93	flavonoids	diosmetin-7-*O*-rutinoside (diosmin)	$C_{28}H_{32}O_{15}$	607.16684	/	/	/
94	flavonoids	cirsimarin	$C_{23}H_{24}O_{11}$	475.12461	/	/	/
95	flavonoids	hispidulin-7-rutinoside/isomers	$C_{28}H_{32}O_{15}$	607.16684	/	/	13.82

Nr.	Phytochemical class	Compound	Molecular formula	Adduct Ion (*m*/*z*) Monitored Negative Ion	Retention time (min)		
					SE	GE	CE
96	flavonoids	hispidulin-*O*-hexoside/ isomers	$C_{22}H_{22}O_{11}$	461.10893	14.98	13.63/14.96	13.64
97	flavonoids	hispidulin	$C_{16}H_{12}O_6$	299.05614	/	/	/
98	flavonoids	gallocatechin/epigallocatechin	$C_{15}H_{14}O_7$	305.06668	/	5.47	15.73
99	flavonoids	luteolin	$C_{15}H_{10}O_6$	285.04049	/	/	/
100	flavonoids	quecetin-3-*O* glucuronid	$C_{21}H_{18}O_{13}$	477.06749	/	/	/
101	flavonoids	apigenin-7-rutinoside	$C_{27}H_{30}O_{14}$	577.15630	/	/	13.49
102	flavonoids	narirutin (naringenin-7-*O* rutinoside)	$C_{27}H_{32}O_{14}$	579.17195	/	12.24	/
103	flavonoids	apigenin-7-*O*-glucuronide	$C_{21}H_{18}O_{11}$	445.07763	/	/	/
104	flavonoids	procyanidine B1/B2	$C_{30}H_{26}O_{12}$	577.13515	/	/	/
105	flavonoids	procyanidine	$C_{30}H_{26}O_{13}$	593.13006	16	/	/
106	flavonoids	diosmetin	$C_{16}H_{12}O_6$	299.05611	/	16.93	16.92
107	flavonoids	isoquercitrin/quercitrin (quercetin-3-rhamnoside)	$C_{21}H_{20}O_{11}$	447.09331	13.8	13.76/14.66	13.75
108	flavonoids	quercetin-3-*O* rutinoside	$C_{33}H_{40}O_{21}$	771.19896	/	10.25	10.37
109	flavonoids	isorhamnetin-3-*O*-rutinosid	$C_{28}H_{32}O_{16}$	623.16178	14	14	13.96
110	flavonoids	isorhamentin-3-*O*-hexoside	$C_{22}H_{22}O_{12}$	477.10387	13.9	13.87	11.97/13.86
111	flavonoids	quercetin-acetyl-hexoside	$C_{23}H_{22}O_3$	345.14964	/	/	/
112	flavonoids	epigallocatechin gallate	$C_{22}H_{18}O_{11}$	457.07766	/	/	/
113	flavonoids	amentoflavone	$C_{30}H_{18}O_{10}$	537.08274	/	18.77	/
114	flavonoids	quercetin-3-(6-malonyl)-hexoside	$C_{24}H_{22}O_{15}$	549.08862	13.09	/	/
115	flavonoids	quercetagetin (6-hydroxyquercetin)	$C_{15}H_{10}O_8$	317.03032	/	/	7.82
116	flavonoids	phlorizin	$C_{21}H_{24}O_{10}$	435.12969	/	/	10.27
117	flavonoids	chalcone	$C_{15}H_{12}O$	207.08156	/	/	/
118	flavonoids	robinin	$C_{33}H_{40}O_{19}$	739.20913	12.42	12.39	12.67
119	flavonoids	tomentosanol E	$C_{30}H_{36}O_7$	507.23885	/	/	/
120	flavonoids	tomentosanol D	$C_{20}H_{20}O_6$	355.11874	/	/	/
121	flavonoids	vitexin-2-*O*-rhamnoside	$C_{27}H_{30}O_{14}$	577.15630	/	/	14.29
122	flavonoids	sophoraflavanone G/ isomers	$C_{25}H_{28}O_6$	423.18134	/	19.48	24.22
123	flavonoids	leachianone A	$C_{26}H_{30}O6$	437.19699	/	/	/
124	flavonoids	lehmannin	$C_{25}H_{28}O_5$	407.18642	/	/	/
125	flavonoids	kushenol/ leachianone isomers	$C_{25}H_{28}O_5$	407.18642	/	/	/
126	flavonoids	prostatrol F	$C_{25}H_{28}O_4$	391.19151	/	/	/
127	flavonoids	prostatrol C	$C_{21}H_{22}O_5$	353.13947	/	/	/
128	flavonoids	alopecurone A	$C_{39}H_{38}O_9$	649.24433	/	/	/
129	flavonoids	sophoronol A	$C_{20}H_{18}O_7$	369.09800	/	/	/
130	flavonoids	sophoronol B	$C_{21}H_{20}O_8$	399.10857	/	/	/
131	flavonoids	2′-hydroxygenistein	$C_{15}H_{10}O_6$	285.04049	/	/	/
132	flavonoids	ginkgetin	$C_{32}H_{22}O_{10}$	565.11404	/	21.39	21.37
133	flavonoids	daphnin	$C_{15}H_{16}O_9$	339.072155	5.16	5.18	/
134	flavonoids	morachalcone	$C_{20}H_{20}O_5$	339.1237966	/	/	/
135	flavonoids	cyanidin-3-*O*-hexoside	$C_{21}H_{21}ClO_{11}$	483.0699619	/	/	/
136	monoterpenes	albiflorin	$C_{23}H_{28}O_{11}$	479.15591	/	/	/
137	polyholosides	inulin	$C_{18}H_{34}O_{17}$	521.17235	/	/	/
138	sesquiterpenes	abscisic acid	$C_{15}H_{20}O_4$	263.12891	14.87	/	14.84
139	sesquiterpenes	cynaropicrin	$C_{19}H_{22}O_6$	345.13439	/	11.5	/
140	sesquiterpenes	lactucopicrin	$C_{23}H_{22}O_7$	409.12930	7.67	/	/
141	sesquiterpenes	lactucin	$C_{15}H_{16}O_5$	275.09252	/	/	/
142	sesquiterpenes	valerenic acid	$C_{15}H_{22}O_2$	233.15473	/	/	/
143	triterpenes	betulin	$C_{30}H_{50}O_2$	441.37383	/	/	/
144	triterpenes	oleanolic acid	$C_{30}H_{48}O_3$	455.35309	/	/	/
145	triterpenes	ursolic acid	$C_{30}H_{48}O_3$	455.35307	/	/	/
146	triterpenes	taraxasterol	$C_{30}H_{48}O$	423.36326	/	/	/

Nr.	Phytochemical class	Compound	Molecular formula	Adduct Ion (*m/z*) Monitored Negative Ion	Retention time (min)		
					SE	GE	CE
147	triterpenes	taraxerol	$C_{30}H_{50}O$	425.37889	/	/	/
148	triterpenes	spicoside A	$C_{30}H_{26}O_{15}$	625.11992	/	/	/
149	triterpenes	taraxerone	$C_{30}H_{48}O$	423.36324	/	/	/
150	triterpenes	calenduloside G/isomers	$C_{42}H_{66}O_{14}$	793.43800	21.02/23.03	/	22.99
151	triterpenes	calenduloside E/isomers	$C_{36}H_{56}O_{9}$	631.38518	23.47	/	/
152	triterpenes	calenduloside F/isomers	$C_{42}H_{66}O_{14}$	793.43800	/	/	/

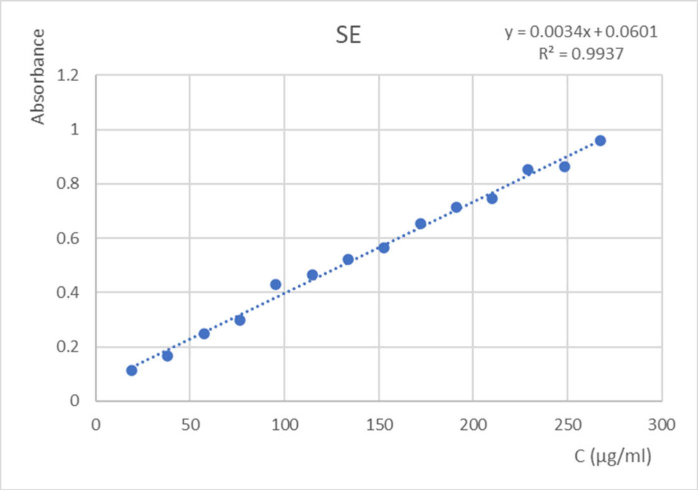

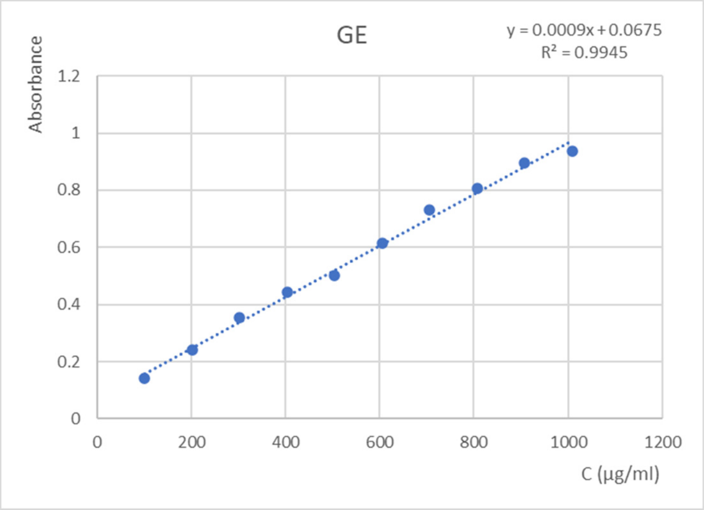

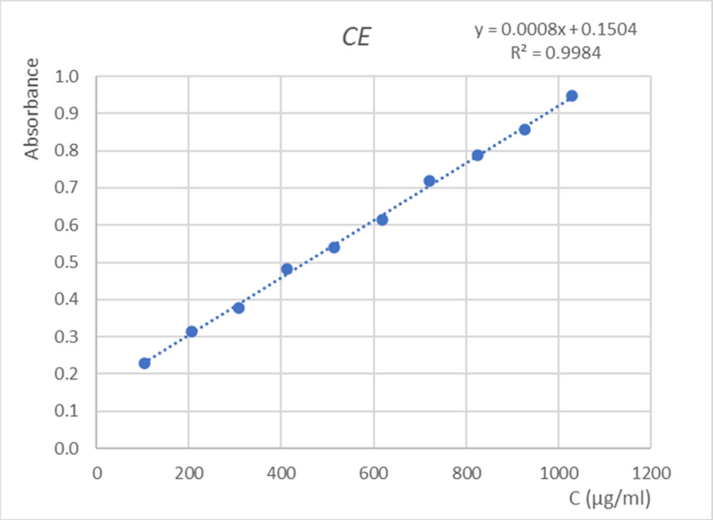

Figure S32. Regression trendlines and equations – FRAP assay (SE = *Sophorae flos* extract; GE = *Ginkgo bilobae folium* extract; CE = *Calendulae flos* extract).

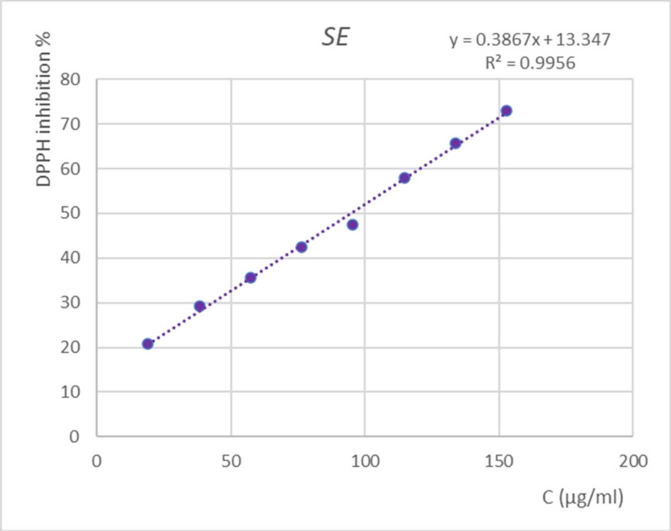

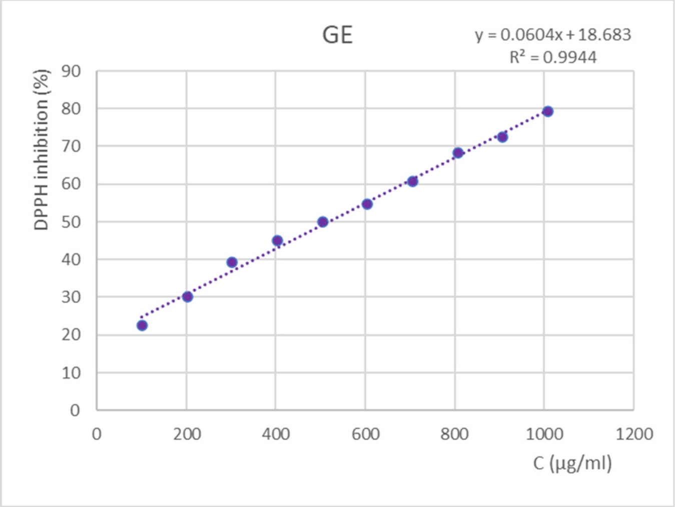

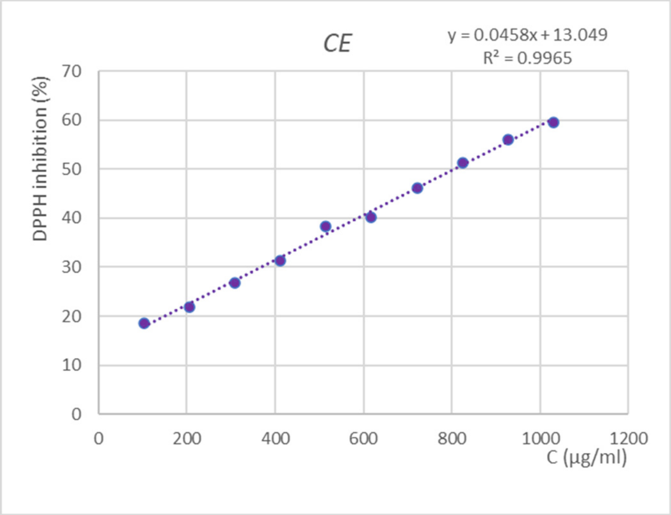

Figure S33. Regression trendlines and equations – DPPH assay (SE = *Sophorae flos* extract; GE = *Ginkgo bilobae folium* extract; CE = *Calendulae flos* extract).

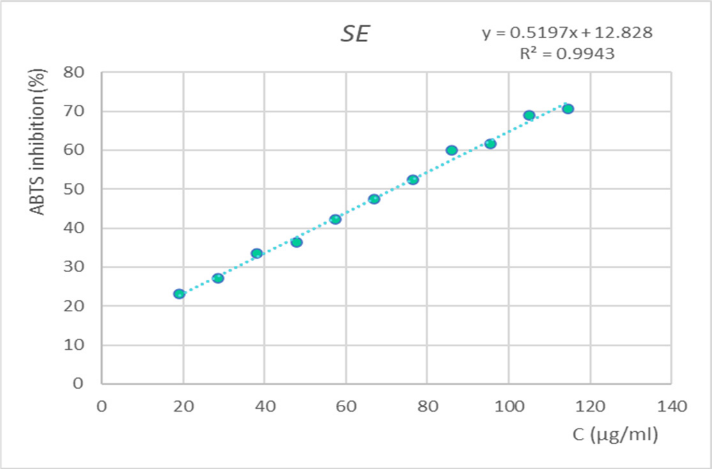

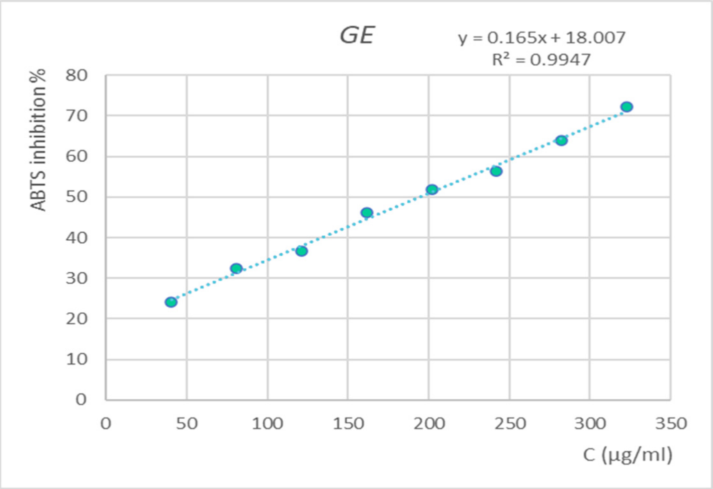

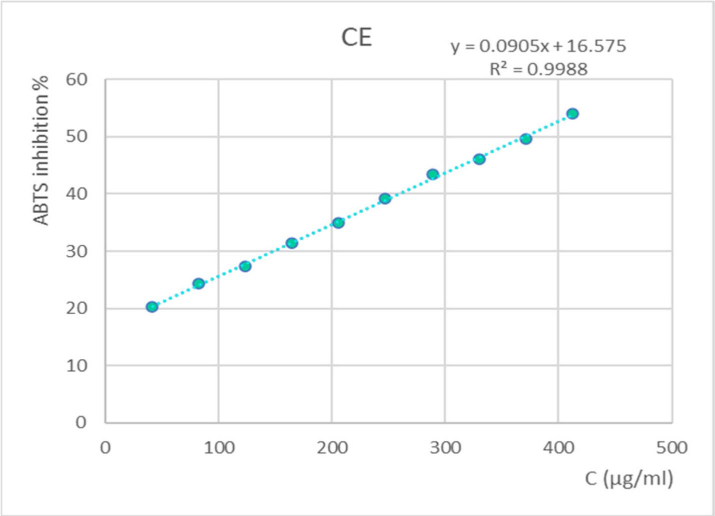

Figure S34. Regression trendlines and equations – ABTS assay (SE = *Sophorae flos* extract; GE = *Ginkgo bilobae folium* extract; CE = *Calendulae flos* extract).

Table S2. Correlation between flavonoid content (FLV) and antioxidant methods.

		ABTS (log_{10})	DPPH(log_{10})	FRAP(log_{10})	FLV
ABTS (log_{10})	Pearson correlation	1.000	0.979	0.894	0.924
	Sig.(2-tailed)	/	0.129	0.296	0.249
DPPH (log_{10})	Pearson correlation	0.979	1.000	0.966	0.982
	Sig.(2-tailed)	0.129	/	0.167	0.120
FRAP (log_{10})	Pearson correlation	0.894	0.966	1.000	0.997
	Sig.(2-tailed)	0.296	0.167	/	0.047*
FLV	Pearson correlation	0.924	0.982	0.997	1.000
	Sig.(2-tailed)	0.249	0.120	0.047*	/

*significant at 0.05 level

Table S3. Correlation between phenolcarboxylic acids content (PCA) and antioxidant methods.

		ABTS (log_{10})	DPPH(log_{10})	FRAP(log_{10})	PCA
ABTS (log_{10})	Pearson correlation	1.000	0.979	0.894	0.878
	Sig.(2-tailed)	/	0.129	0.296	0.318
DPPH (log_{10})	Pearson correlation	0.979	1.000	0.966	0.956
	Sig.(2-tailed)	0.129	/	0.167	0.189
FRAP (log_{10})	Pearson correlation	0.894	0.966	1.000	0.999
	Sig.(2-tailed)	0.296	0.167	/	0.022
PCA	Pearson correlation	0.878	0.956	0.999*	1.000
	Sig.(2-tailed)	0.318	0.189	0.022	/

*significant at 0.05 level

Table S4. Correlation between polyphenolic content (PPC) and antioxidant methods.

		ABTS (log_{10})	DPPH(log_{10})	FRAP(log_{10})	PPC
ABTS (log_{10})	Pearson correlation	1.000	0.979	0.894	0.952
	Sig.(2-tailed)	/	0.129	0.296	0.198
DPPH (log_{10})	Pearson correlation	0.979	1.000	0.966	0.994
	Sig.(2-tailed)	0.129	/	0.167	0.068
FRAP (log_{10})	Pearson correlation	0.894	0.966	1.000	0.988
	Sig.(2-tailed)	0.296	0.167	/	0.099
PPC	Pearson correlation	0.952	0.994	0.988	1.000
	Sig.(2-tailed)	0.198	0.068	0.099	/

* significant at 0.05 level

Table S5. Correlation between UHPLC-HRMS/MS method (QHPLC), antioxidant methods and spectrophotometric methods.

		QHPLC
ABTS (log_{10})	Pearson correlation	0.896
	Sig.(2-tailed)	0.292
DPPH (log_{10})	Pearson correlation	0.967
	Sig.(2-tailed)	0.163
FRAP (log_{10})	Pearson correlation	1.000
	Sig.(2-tailed)	0.004*
PPC	Pearson correlation	0.989
	Sig.(2-tailed)	0.095
PCA	Pearson correlation	0.999
	Sig.(2-tailed)	0.026*

		QHPLC
**FLV**	Pearson correlation	0.998
	Sig.(2-tailed)	0.043*
**QHPLC**	Pearson correlation	1.000
	Sig.(2-tailed)	/

* significant at 0.05 level

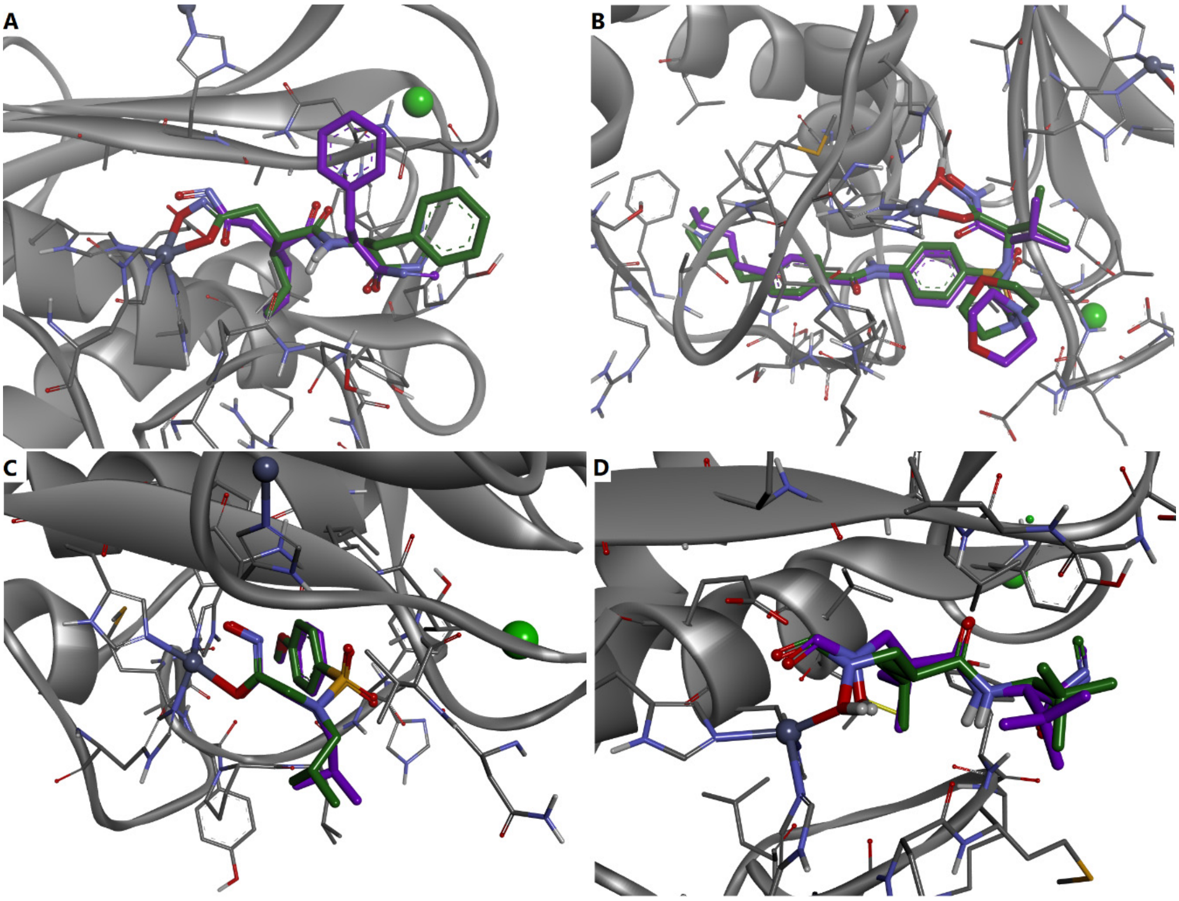

Figure S35. Superposition of predicted conformations for MMPs inhibitors on experimentally determined conformations. A – MMP1 inhibitor PLH, B – MMP2 inhibitor SC-74020; C – MMP3 inhibitor NGH; D – MMP9 inhibitor NFH.

Table S6. Molecular docking results after screening the selected phytochemicals.

	**MMP1**		**MMP2**		**MMP3**		**MMP9**	
**Compound**	**ΔG (kcal/mol)**	**LE**	**ΔG (kcal/mol)**	**LE**	**ΔG (kcal/mol)**	**LE**	**ΔG (kcal/mol)**	**LE**
bilobalide	−6.076	0.2642	−7.183	0.3123	−6.522	0.2836	−5.844	0.2541
bilobetin	−9.194	0.2242	−9.645	0.2352	−9.334	0.2277	−9.347	0.2280
caffeic acid	−7.677	0.5905	−7.303	0.5618	−7.256	0.5582	−8.245	0.6342
calendoflavoside	−8.576*	0.1949	−7.401*	0.1682	−7.860	0.1786	−7.629*	0.1734
calenduloside E	−7.911	0.1758	−9.330	0.2073	−7.574*	0.1683	−7.771*	0.1727

calenduloside F	–7.688	0.1373	–8.626	0.1540	–7.513*	0.1342	–7.467*	0.1333
calenduloside G	–8.383*	0.1497	–9.923*	0.1772	–8.384*	0.1497	–9.655*	0.1724
calenduloside H	–7.565	0.1129	–8.852*	0.1321	–8.367	0.1249	–8.395	0.1253
catechin	–8.679	0.4133	–7.853	0.3740	–7.864*	0.3745	–9.748	0.4642
chlorogenic acid	–8.912*	0.3565	–9.512	0.3805	–8.807	0.3523	–10.665	0.4266
fisetin	–9.189	0.4376	–8.756	0.4170	–8.403	0.4001	–9.103	0.4335
ginkgolide A	–7.329	0.2527	–7.579	0.2613	–8.244	0.2843	–7.362*	0.2539
ginkgolide B	–6.495	0.2165	–7.667*	0.2556	–7.105	0.2368	–6.818*	0.2273
ginkgolide C	–6.360	0.2052	–8.121	0.2620	–7.097*	0.2289	–6.953*	0.2243
isoquercitrin	–8.529*	0.2585	–8.215	0.2489	–8.060*	0.2442	–8.760	0.2655
isorhamnetin	–8.303	0.3610	–7.356	0.3198	–7.881	0.3427	–8.680	0.3774
kaempferol	–8.342	0.3972	–8.288	0.3947	–8.001	0.3810	–8.627	0.4108
kaikasaponin I	–8.541	0.1553	–8.625	0.1568	–8.884	0.1615	–7.896*	0.1436
kaikasaponin III	–8.400*	0.1292	–9.848*	0.1515	–8.615*	0.1325	–8.162	0.1256
narcissin	–8.975*	0.2040	–7.919*	0.1800	–8.865*	0.2015	–8.294*	0.1885
quercetin	–9.280	0.4218	–8.694	0.3952	–8.508	0.3867	–8.944	0.4065
rhamnetin	–9.470	0.4117	–8.275	0.3598	–8.642	0.3757	–9.184	0.3993
rutin	–8.164	0.1899	–8.054*	0.1873	–9.406*	0.2187	–8.730*	0.2030
sophoricoside	–7.801*	0.2516	–10.655	0.3437	–9.547	0.3080	–11.492*	0.3707
typhaneoside	–8.619	0.1596	–8.506	0.1575	–9.445*	0.1749	–7.595*	0.1406
PLH	–6.467*	0.2587	–	–	–	–	–	–
SC-74020	–	–	–9.306*	0.2327	–	–	–	–
NGH	–	–	–	–	–7.509*	0.3576	–	–
NFH	–	–	–	–	–	–	–6.913*	0.3142

ΔG – binding energy, LE – ligand efficiency, * - presence of metal bonds between the docked compound and catalytic zinc.

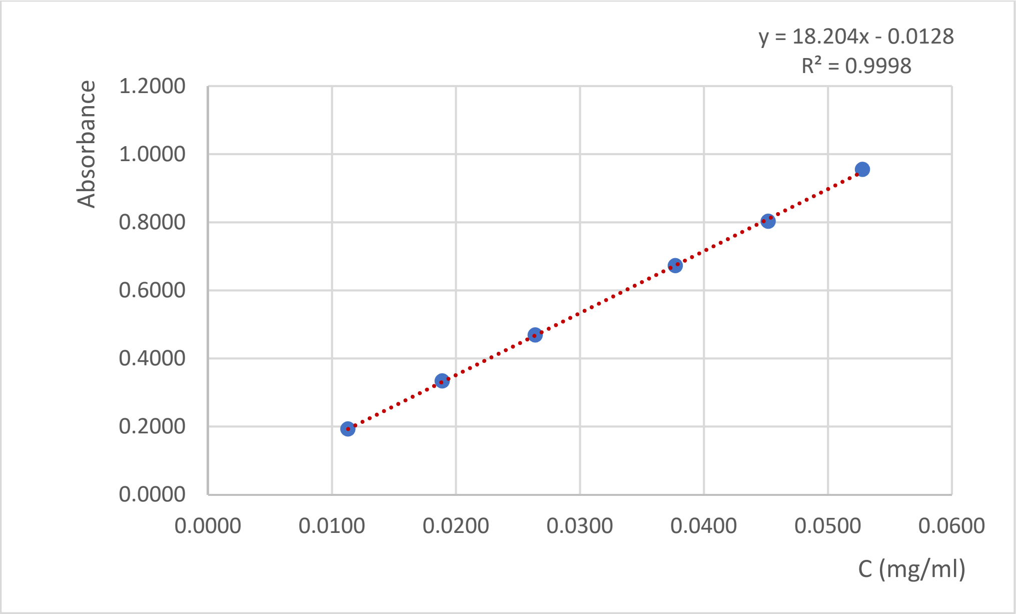

Figure S36. Chlorogenic acid standard curve.

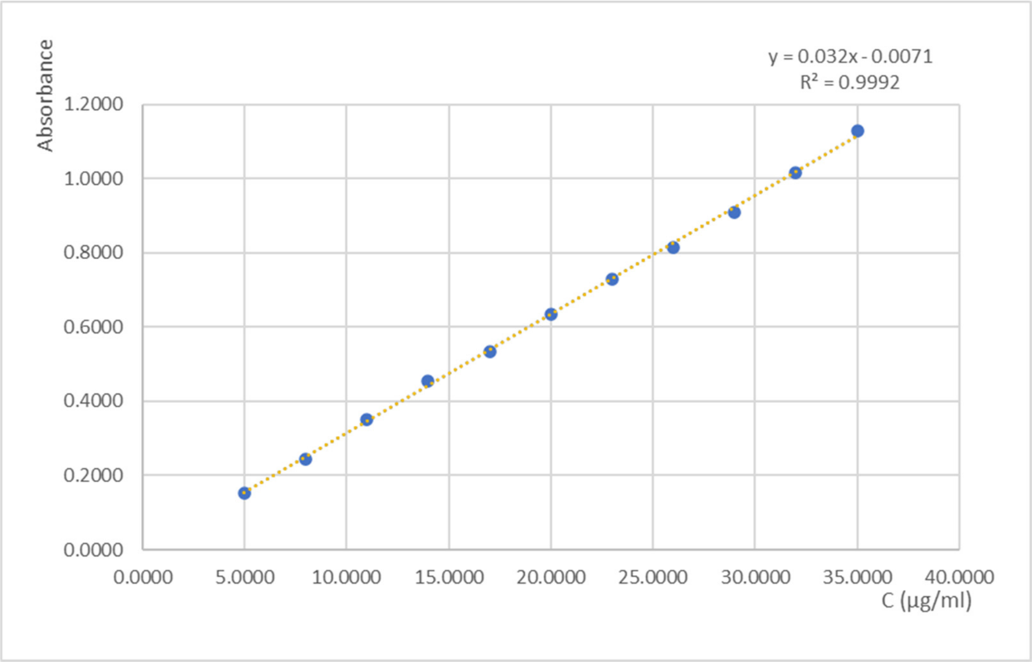

Figure S37. Rutin standard curve.

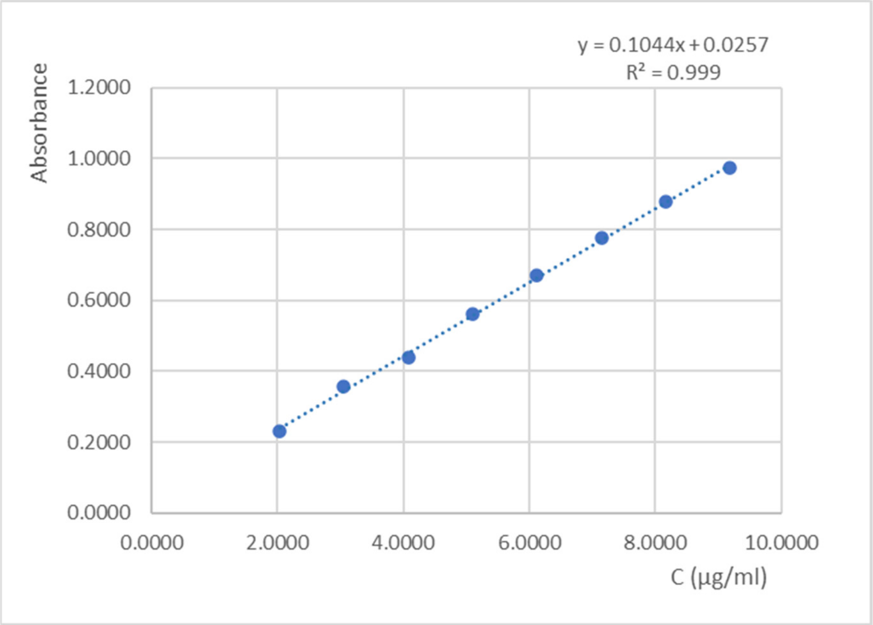

Figure S38. Tannic acid standard curve.
